# Association of *PDGFRA* polymorphisms with the risk of corneal astigmatism in a Japanese population

**DOI:** 10.1038/s41598-023-43333-1

**Published:** 2023-09-26

**Authors:** Hideharu Fukasaku, Akira Meguro, Masaki Takeuchi, Nobuhisa Mizuki, Masao Ota, Kengo Funakoshi

**Affiliations:** 1 Department of Neuroanatomy, Yokohama City University Graduate School of Medicine, Yokohama, Kanagawa 236-0004 Japan; 2 Fukasaku Eye Institute, Yokohama, Kanagawa 220-0003 Japan; 3 Department of Ophthalmology and Visual Science, Yokohama City University Graduate School of Medicine, Yokohama, Kanagawa 236-0004 Japan; 4 Department of Advanced Medicine for Ocular Diseases, Yokohama City University Graduate School of Medicine, Yokohama, Kanagawa 236-0004 Japan; 5 Department of Medicine, Division of Hepatology and Gastroenterology, Shinshu University School of Medicine, Matsumoto, Nagano, 390-8621 Japan

**Keywords:** Genetic association study, Refractive errors

## Abstract

Corneal astigmatism is reportedly associated with polymorphisms of the platelet-derived growth factor receptor alpha (*PDGFRA*) gene region in Asian populations of Chinese, Malay, and Indian ancestry and populations of European ancestry. In this study, we investigated whether these *PDGFRA* polymorphisms are associated with corneal astigmatism in a Japanese population. We recruited 1,535 cases with corneal astigmatism (mean corneal cylinder power across both eyes: ≤  − 0.75 diopters [D]) and 842 controls (> − 0.75 D) to genotype 13 single-nucleotide polymorphisms (SNPs) in the *PDGFRA* gene region. We also performed imputation analysis in the region, with 179 imputed SNPs included in the statistical analyses. The *PDGFRA* SNPs were not significantly associated with the cases with corneal astigmatism ≤  − 0.75 D. However, the odds ratios (ORs) of the minor alleles of SNPs in the upstream region of *PDGFRA*, including rs7673984, rs4864857, and rs11133315, tended to increase according to the degree of corneal astigmatism, and these SNPs were significantly associated with the cases with corneal astigmatism ≤  − 1.25 D or ≤  − 1.50 D (*P*c < 0.05, OR = 1.34–1.39). These results suggest that *PDGFRA* SNPs play a potential role in the development of greater corneal astigmatism.

## Introduction

Astigmatism is a refractive error in which light rays entering the eye are not focused on a single point on the retina due to irregularities in the shape or curvature of the cornea or lens, resulting in blurred vision at all distances^1^. Astigmatism is the most common refractive error worldwide and a recent meta-analysis revealed that the estimated pool prevalences of astigmatism in children and adults throughout the world were 14.9% and 40.4%, respectively^2^. Uncorrected astigmatism in children can affect various visual functions, including grating acuity, vernier acuity, contrast sensitivity, recognition acuity, and stereoacuity, and is associated with the development of amblyopia and myopia^3–7^. Therefore, early detection and proper treatment of astigmatism, especially severe astigmatism, in children are important to prevent future visual impairment. The etiology of astigmatism remains unclear, but both environmental and genetic factors are thought to contribute to the development of astigmatism^1,8^.

Corneal astigmatism, which is caused by irregularities in the cornea’s shape or curvature, is the most common type of astigmatism. Corneal astigmatism is reportedly associated with single-nucleotide polymorphisms (SNPs) of the platelet-derived growth factor receptor alpha (*PDGFRA*) gene region on chromosome 4q12. Fan et al. performed the first genome-wide association study (GWAS) of corneal astigmatism with Asian ancestry cohorts and initially reported that corneal astigmatism was significantly associated with *PDGFRA* SNPs (lead SNP: rs7677751)^9^. Subsequently, the association of the *PDGFRA* gene region with corneal astigmatism was replicated in a UK European ancestry cohort, and rs6554163 was associated with corneal astigmatism^10^. In addition, recent large-scale GWASs of corneal astigmatism with European and/or Asian ancestry cohorts provided strong evidence of association between corneal astigmatism and *PDGFRA* SNPs (lead SNPs: rs7673984 or rs4864857)^11,12^, suggesting that *PDGFRA* plays a key role in the development of corneal astigmatism through genetic polymorphisms. However, another study showed no evidence for replication of the *PDGFRA* gene region in an Australian cohort of European ancestry^13^. Thus, further genetic studies are needed to clarify the contribution of the *PDGFRA* gene region to the development of corneal astigmatism.

To date, the association between the *PDGFRA* gene region and corneal astigmatism has been assessed in individuals of Chinese, Malay, and Indian ancestry among Asians, but not yet in individuals of Japanese ancestry. This study aimed to investigate whether SNPs in the *PDGFRA* region are associated with the risk of corneal astigmatism in a Japanese population. Here, we performed a comprehensive association analysis of SNPs in the *PDGFRA* gene region among Japanese patients with corneal astigmatism.

## Results

### Demographic and characteristics of the study populations

According to previous studies with Asian populations^9,11^, we defined individuals with mean corneal cylinder power ≤  − 0.75 diopters (D) across both eyes as corneal astigmatism cases (n = 1535), while controls were defined as mean corneal cylinder power >  − 0.75 D across both eyes (n = 842). The demographic and clinical characteristics of the cases and controls are shown in Table 1. A total of 40.1% of cases and 44.5% of controls were male, and the mean ages of the cases and controls were 43.6 ± 15.4 years and 53.5 ± 13.5 years, respectively. The average corneal cylinder powers of the cases and controls were − 1.33 ± 0.54 D and − 0.43 ± 0.17 D, respectively.

**Table 1:** Demographics and characteristics of the study populations.

Characteristic	Cases*	Controls**
Number of participants	1535	842
Male/female, %	40.1/59.9	44.5/55.5
Mean age, year	43.6 ± 15.4	53.5 ± 13.5
Mean corneal cylinder power, diopter (D)	− 1.33 ± 0.54	− 0.43 ± 0.17

*Corneal astigmatism cases defined as mean corneal cylinder power ≤  − 0.75D across both eyes.

**Controls defined as mean corneal cylinder power >  − 0.75D across both eyes.

### Association analysis

We genotyped 13 SNPs in the *PDGFRA* gene region: nine tagging SNPs (rs11133315, rs6554162, rs2303429, rs7656613, rs1547905, rs3816888, rs17739921, rs1826426, and rs6554170) and four SNPs reportedly associated with corneal astigmatism in previous studies (rs7673984, rs4864857, rs6554163 and rs7677751)^9–12^. The observed and expected frequencies of each genotype for the 13 SNPs were in Hardy–Weinberg equilibrium (HWE) (*P* > 0.05) in both the case and control groups.

Table 2 shows the results of the association analysis for the 13 SNPs. No significant association was found for any of the 13 SNPs in the cases with corneal astigmatism ≤  − 0.75 D. On the other hand, when the cases were stratified according to mean corneal cylinder power across both eyes (i.e., ≤  − 1.00 D, ≤  − 1.25 D, and ≤  − 1.50 D), the odds ratios (ORs) of the minor alleles of the seven SNPs rs11133315, rs7673984, rs4864857, rs6554162, rs6554163, rs7677751, and rs1547905 tended to increase according to the degree of corneal astigmatism, and significant associations were found for rs11133315, rs7673984, rs4864857, rs6554163, and rs7677751 in the cases with corneal astigmatism ≤  − 1.25 D (rs11133315: *P*c = 0.026, OR = 1.34; rs7673984 and rs4864857: *P*c = 0.016, OR = 1.38; rs6554163: *P*c = 0.022, OR = 1.37; rs7677751: *P*c = 0.035, OR = 1.35).

**Table 2:** Association results for 13 genotyped SNPs in the *PDGFRA* gene region.

SNP	Position on Chr. 4 (GRCh37)	Alleles (1 > 2)	Phenotype		N	Minor Allele Freq	*P*	*P*c	OR (95% CI)
				Criteria for CA (D)					
rs11133315	55,082,158	G>A	Controls	Mean > − 0.75	842	0.178			
			Cases	Mean ≤ − 0.75	1535	0.196	0.16		1.12 (0.96–1.32)
				Mean ≤ − 1.00	1134	0.209	0.021	0.19	1.23 (1.03–1.47)
				Mean ≤ − 1.25	787	0.220	0.0028	0.026	1.34 (1.11–1.63)
				Mean ≤ − 1.50	514	0.217	0.0043	0.038	1.39 (1.11–1.73)
rs7673984	55,088,761	C>T	Controls	Mean > − 0.75	842	0.156			
			Cases	Mean ≤ − 0.75	1535	0.177	0.079		1.17 (0.98–1.38)
				Mean ≤ − 1.00	1134	0.188	0.012	0.11	1.26 (1.05–1.52)
				Mean ≤ − 1.25	787	0.199	0.0018	0.016	1.38 (1.13–1.69)
				Mean ≤ − 1.50	514	0.194	0.0062	0.055	1.39 (1.10–1.75)
rs4864857	55,089,814	T>C	Controls	Mean > − 0.75	842	0.156			
			Cases	Mean ≤ − 0.75	1535	0.177	0.079		1.17 (0.98–1.38)
				Mean ≤ − 1.00	1134	0.188	0.012	0.11	1.26 (1.05–1.52)
				Mean ≤ − 1.25	787	0.199	0.0018	0.016	1.38 (1.13–1.69)
				Mean ≤ − 1.50	514	0.194	0.0062	0.055	1.39 (1.10–1.75)
rs6554162	55,093,955	G>A	Controls	Mean > − 0.75	842	0.284			
			Cases	Mean ≤ − 0.75	1535	0.301	0.19		1.10 (0.96–1.26)
				Mean ≤ − 1.00	1134	0.313	0.035	0.32	1.18 (1.01–1.37)
				Mean ≤ − 1.25	787	0.328	0.0072	0.065	1.26 (1.06–1.49)
				Mean ≤ − 1.50	514	0.326	0.011	0.095	1.29 (1.06–1.56)
rs6554163	55,102,559	T>A	Controls	Mean > − 0.75	842	0.157			
			Cases	Mean ≤ − 0.75	1535	0.177	0.094		1.16 (0.97–1.37)
				Mean ≤ − 1.00	1134	0.189	0.014	0.13	1.26 (1.05–1.51)
				Mean ≤ − 1.25	787	0.199	0.0025	0.022	1.37 (1.12–1.67)
				Mean ≤ − 1.50	514	0.194	0.0081	0.073	1.37 (1.09–1.73)
rs7677751	55,124,460	C>T	Controls	Mean > − 0.75	842	0.150			
			Cases	Mean ≤ − 0.75	1535	0.173	0.063		1.18 (0.99–1.40)
				Mean ≤ − 1.00	1134	0.182	0.016	0.14	1.26 (1.04–1.51)
				Mean ≤ − 1.25	787	0.192	0.0039	0.035	1.35 (1.10–1.66)
				Mean ≤ − 1.50	514	0.186	0.0098	0.088	1.37 (1.08–1.73)
rs2303429	55,124,870	C>T	Controls	Mean > − 0.75	842	0.111			
			Cases	Mean ≤ − 0.75	1535	0.107	0.44		0.92 (0.76–1.13)
				Mean ≤ − 1.00	1134	0.103	0.091		0.83 (0.66–1.03)
				Mean ≤ − 1.25	787	0.102	0.062		0.79 (0.62–1.01)
				Mean ≤ − 1.50	514	0.108	0.13		0.81 (0.61–1.07)
rs7656613	55,141,843	T>C	Controls	Mean > − 0.75	842	0.464			
			Cases	Mean ≤ − 0.75	1535	0.460	0.93		1.01 (0.89–1.14)
				Mean ≤ − 1.00	1134	0.458	0.66		1.03 (0.90–1.18)
				Mean ≤ − 1.25	787	0.449	0.61		1.04 (0.89–1.21)
				Mean ≤ − 1.50	514	0.455	0.36		1.08 (0.91–1.29)
rs1547905	55,146,754	C>A	Controls	Mean > − 0.75	842	0.145			
			Cases	Mean ≤ − 0.75	1535	0.160	0.21		1.12 (0.94–1.33)
				Mean ≤ − 1.00	1134	0.170	0.059		1.20 (0.99–1.44)
				Mean ≤ − 1.25	787	0.179	0.033	0.30	1.25 (1.02–1.54)
				Mean ≤ − 1.50	514	0.178	0.038	0.34	1.29 (1.01–1.63)
rs3816888	55,146,761	C>T	Controls	Mean > − 0.75	842	0.021			
			Cases	Mean ≤ − 0.75	1535	0.022	0.69		1.09 (0.71–1.69)
				Mean ≤ − 1.00	1134	0.021	0.56		1.15 (0.72–1.85)
				Mean ≤ − 1.25	787	0.020	0.54		1.18 (0.69–2.03)
				Mean ≤ − 1.50	514	0.020	0.55		1.17 (0.70–1.98)
rs17739921	55,164,866	A>C	Controls	Mean > − 0.75	842	0.285			
			Cases	Mean ≤ − 0.75	1535	0.275	0.35		0.94 (0.81–1.07)
				Mean ≤ − 1.00	1134	0.275	0.32		0.93 (0.80–1.08)
				Mean ≤ − 1.25	787	0.274	0.22		0.90 (0.76–1.06)
				Mean ≤ − 1.50	514	0.264	0.079		0.84 (0.69–1.02)
rs1826426	55,167,287	A>G	Controls	Mean > − 0.75	842	0.403			
			Cases	Mean ≤ − 0.75	1535	0.411	0.21		1.08 (0.96–1.23)
				Mean ≤ − 1.00	1134	0.416	0.061		1.14 (0.99–1.31)
				Mean ≤ − 1.25	787	0.402	0.31		1.08 (0.93–1.27)
				Mean ≤ − 1.50	514	0.400	0.27		1.11 (0.92–1.32)
rs6554170	55,174,885	C>T	Controls	Mean > − 0.75	842	0.357			
			Cases	Mean ≤ − 0.75	1535	0.366	0.22		1.08 (0.95–1.23)
				Mean ≤ − 1.00	1134	0.368	0.13		1.11 (0.97–1.28)
				Mean ≤ − 1.25	787	0.352	0.59		1.04 (0.89–1.22)
				Mean ≤ − 1.50	514	0.349	0.57		1.05 (0.88–1.27)

*SNP* single-nucleotide polymorphism, *1* major allele, *2* minor allele, *CA* corneal astigmatism, *Pc* corrected *P*-value, *OR* odds ratio, *CI* confidence interval.

The highest ORs for these seven SNPs were found in the cases with corneal astigmatism ≤  − 1.50 D (rs11133315: OR = 1.39; rs7673984: OR = 1.39; rs4864857: OR = 1.39; rs6554162: OR = 1.29; rs6554163: OR = 1.37; rs7677751: OR = 1.37; rs1547905: OR = 1.29), however, of these ORs, only the OR for rs11133315 reached significance (*P*c = 0.038) due to the limited sample size of the cases with corneal astigmatism ≤  − 1.50 D. These seven SNPs, especially rs11133315, rs7673984, rs4864857, rs6554163, and, rs7677751, were in strong linkage disequilibrium (LD) with each other, but not with the other six SNPs (Supplementary Fig. S1).

### Imputation analysis

**Figure 1:**
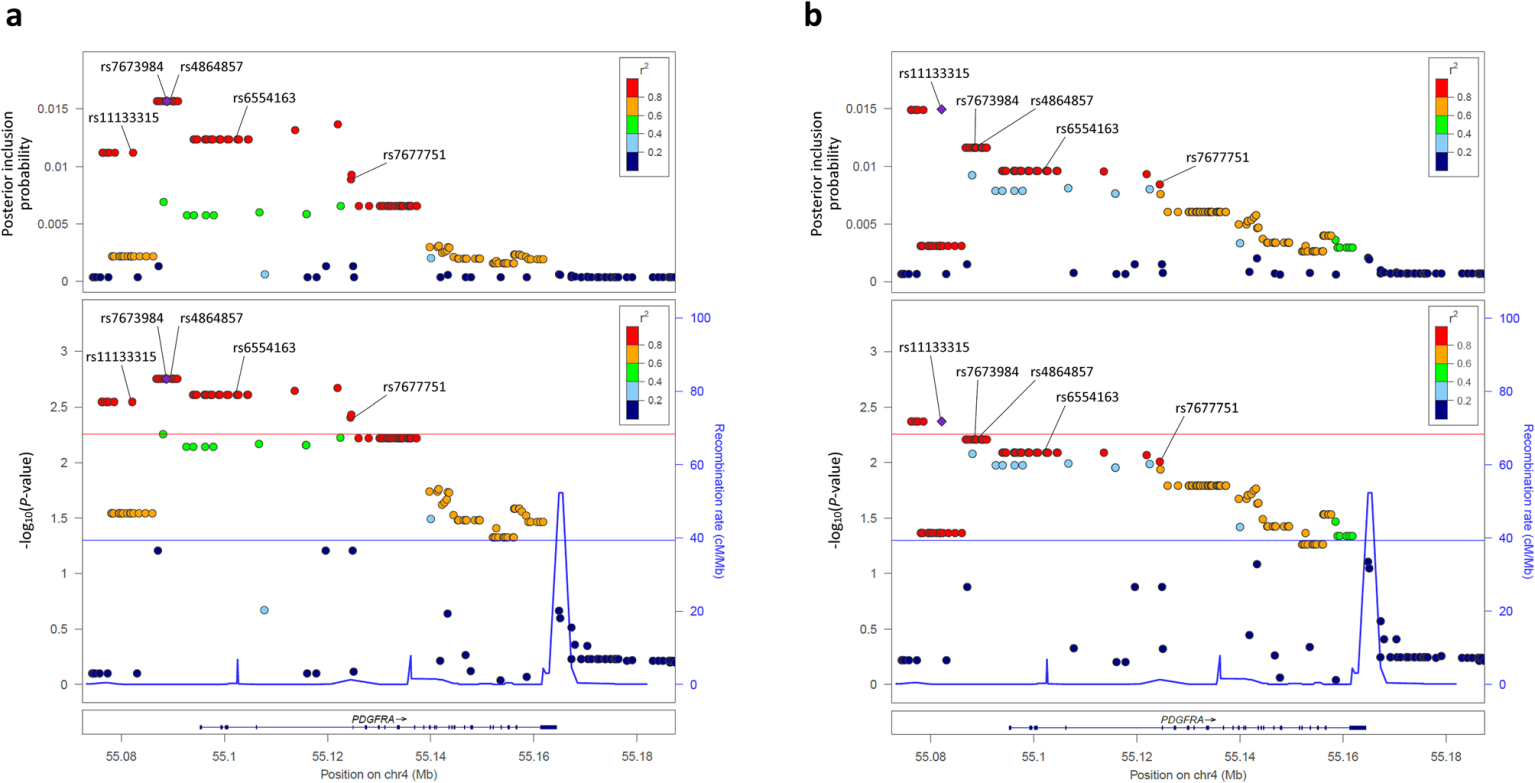
In-depth SNP analysis of the *PDGFRA* gene region in the Japanese cohort. Data are shown for the cases with corneal astigmatism (**a**) ≤  − 1.25 D or (**b**) ≤  − 1.50 D. (*Top row*) Posterior inclusion probability for each SNP genotyped and imputed in the current study. (*Middle row*) Regional association plot for each SNP. The left y-axes represent the − log10 *P* values for associations with corneal astigmatism; the right y-axes represent the estimated recombination rate. The horizontal blue and red lines indicate *P* = 0.05 and *P*c = 0.05 (i.e. *P* = 0.05/9), respectively. One of the lead SNPs in each of the cases with corneal astigmatism ≤  − 1.25 D and ≤  − 1.50 D, (**a**) rs7673984 and (**b**) rs11133315, respectively, is depicted as a purple diamond. The color coding for all other SNPs indicates linkage disequilibrium with (**a**) rs7673984 or (**b**) rs11133315 as follows: red, *r*^2^ ≥ 0.8; yellow, 0.6 ≤ *r*^2^ < 0.8; green, 0.4 ≤ *r*^2^ < 0.6; cyan, 0.2 ≤ *r*^2^ < 0.4; and blue, *r*^2^ < 0.2. (*Bottom row*) Gene annotations.

We performed imputation analysis based on the 13 genotyped SNPs to evaluate potential associations with un-genotyped SNPs in the *PDGFRA* gene region and successfully imputed 179 SNPs in the region. Figure 1a and Supplementary Table S1 show the results of the association analysis for a total of 192 SNPs (13 genotyped and 179 imputed) in the cases with corneal astigmatism ≤  − 1.25 D and controls. The strongest significant association was observed for 15 SNPs, including rs7673984 and rs4864857, which are located within 10 kb upstream of *PDGFRA* (*P*c = 0.016, OR = 1.38). These 15 SNPs were in complete LD (*r*^2^ = 1.0) with each other. The other 32 SNPs, including rs11133315 and rs7677751, in strong LD with these 15 SNPs (*r*^2^ ≥ 0.87) also exhibited significant association with corneal astigmatism (*P*c < 0.05, OR = 1.34–1.37). For the cases with corneal astigmatism ≤  − 1.50 D, a significant association was observed for six SNPs which are located between 13 and 20 kb upstream of *PDGFRA*, including rs11133315 (*P*c = 0.038, OR = 1.39) (Fig. 1b and Supplementary Table S2). For the cases with corneal astigmatism ≤  − 0.75 D or ≤  − 1.00 D, no significant association was observed for any of the 192 tested SNPs (Supplementary Tables S3 and S4).

To identify the most plausible SNPs in the *PDGFRA* gene region, we performed a fine-mapping analysis with FINEMAP^14^. For the cases with corneal astigmatism ≤  − 1.25 D, the fine mapping indicated that the 15 top SNPs including rs7673984 and rs4864857 had the highest posterior inclusion probability (0.016) of being the causal variant at the region (Fig. 1a and Supplementary Table S1). For the cases with corneal astigmatism ≤  − 1.50 D, the six top SNPs, including rs11133315, had the highest posterior inclusion probability (0.015; Fig. 1b and Supplementary Table S2). Additionally, when the stepwise regression analysis adjusted for any of these 21 SNPs was performed, none of other SNPs exhibited any association with the disease (*P* > 0.05), suggesting that any of these 21 SNPs located upstream of *PDGFRA* could account for the association of other SNPs with the disease.

### Functional analysis

The HaploReg database^15^ predicted that three of these 21 SNPs (rs11133315, rs7698425, and rs7681399) would be located within enhancer histone marks and one (rs11133315) in the DNase hypersensitivity region. The database also predicted that 18 of these 21 SNPs would alter the regulatory motif of transcription factors (Supplementary Table S5). Thus, most of these 21 SNPs have the potential to affect the transcriptional regulation of *PDGFRA*. On the other hand, the RegulomeDB database^16^ indicated minimal evidence of transcription factor binding at these 21 SNPs (RegulomeDB score = 4–7), with the highest likelihood of having a regulatory function observed at rs11133315 and rs7673625 (RegulomeDB score = 4; Supplementary Table S5). In addition, the GTEx Portal database^17^ did not show any significant expression quantitative trait loci (eQTLs) for these 21 SNPs in any of the analyzed tissues.

## Discussion

The aim of this study was to assess whether polymorphisms in the *PDGFRA* gene region affected the development of corneal astigmatism in our Japanese population. We performed a comprehensive association analysis of SNPs in the *PDGFRA* gene region among Japanese patients with corneal astigmatism. We found that the association between multiple SNPs located upstream of *PDGFRA* (e.g., rs7673984, rs4864857, and rs11133315) and corneal astigmatism became stronger with the degree of corneal astigmatism and that these SNPs were significantly associated with Japanese patients with higher corneal astigmatism. These findings suggest that *PDGFRA* SNPs play a potential role in the development of greater corneal astigmatism.

*PDGFRA* encodes the alpha isoform of the platelet-derived growth factor (PDGF) receptor, which is a cell-surface receptor tyrosine kinase^18^. PDGFRA initiates various signaling pathways, including Ras-MAPK, Akt/PKB, JNK/SAPK, and PKC, after binding to ligand PDGFs^19^. PDGFRA signaling is associated with many physiological processes, such as embryonic development, cell proliferation, differentiation, migration, and survival, and is critical for organogenesis, including the development of the lung, intestine, skin, testis, and kidney^19,20^. *PDGFRA* signaling also plays a critical function in the development of ocular tissues such as the cornea, lens, and optic nerve^19,21,22^. Several studies have reported that its signaling is involved in corneal fibroblast migration and proliferation, which are fundamental processes for corneal wound healing^23–26^. In addition, treatment with PDGF induces cell elongation in rabbit corneal keratocytes^27^. Similarly, human corneal stromal cells treated with PDGF have a very elongated spindle shape^28^. Fan et al. identified *PDGFRA* as a novel susceptibility locus for corneal astigmatism and raised the possibility that *PDGFRA* genetic polymorphisms may affect the regulation of corneal biometrics, leading to the development of corneal astigmatism^9^. *PDGFRA* genetic polymorphisms are also reportedly associated with variation in corneal curvature^10,29–31^. Thus, taken together, these previous findings suggest that *PDGFRA* affects corneal size and shape through genetic polymorphisms.

In the GWAS meta-analysis with Asian (Chinese, Malay, and Indian living in Singapore) ancestry cohorts (n = 8513) by Fan et al., the *PDGFRA* intronic SNP, rs7677751, showed the strongest association with corneal astigmatism, and its minor allele T was associated with an increased risk (OR = 1.26) of the disease in the cases with corneal astigmatism ≤  − 0.75 D^9^. The association of the *PDGFRA* gene region with corneal astigmatism (≤ − 0.75 D) was replicated in a UK European ancestry cohort (n = 1968), and the minor allele T of another intronic SNP, rs6554163, showed the strongest association with an increased risk (OR = 1.24) of the disease^10^. The association of the *PDGFRA* gene region was also replicated in a larger GWAS meta-analysis of cohorts of Asian ancestry (Chinese living in Beijing, China, and Chinese, Malay and Indian living in Singapore) (n = 9120) and European ancestry (n = 22,250). The minor allele T of rs7673984 showed the strongest association with an increased risk of corneal astigmatism (≤ − 0.75 D) (OR = 1.15 in Asians, 1.11 in Europeans, and 1.12 in the meta-analysis of Asians and Europeans)^11^. Furthermore, a large-scale GWAS for corneal astigmatism analyzed as a continuous trait with individuals of European ancestry from the UK Biobank (n = 86,335) reported a strong association between corneal astigmatism and *PDGFRA* and the minor allele C of a lead SNP, rs4864857, which contributed to an increased risk of the disease (effect size = 0.017)^12^. These four SNPs, rs7677751, rs6554163, rs7673984, and rs4864857, are in strong LD with each other (*r*^2^ ≥ 0.943 in East Asians;* r*^2^ ≥ 0.637 in Europeans)^32^. In our present study with a Japanese cohort, the ORs for minor alleles of these four SNPs in the cases with corneal astigmatism of ≤  − 0.75 D were 1.16 to 1.18, which are similar to those reported in the previous studies^9–11^ but not significant, due to the smaller sample size of the present study compared with those of the previous studies. Moreover, the present study found that the ORs of minor alleles of these SNPs increased with the degree of corneal astigmatism and were significant in the cases with corneal astigmatism of ≤  − 1.25 D, with ORs of 1.35 to 1.38. In our Japanese cohort, the strongest association with the disease was observed in multiple SNPs in complete LD located within 10 kb upstream of *PDGFRA* (*e.g*., rs7673984 and rs4864857) in the cases with corneal astigmatism of ≤  − 1.25 D. Considering the above, the minor allele(s) of rs7677751, rs6554163, rs7673984, and rs4864857 and/or other SNPs in strong LD with these four SNPs may contribute to the development of corneal astigmatism.

On the other hand, Yazar et al. reported no strong evidence of association between *PDGFRA* SNPs and corneal astigmatism in a GWAS with an Australian cohort (n = 1013) of European ancestry, whereas weak association signals were observed in the upstream region of *PDGFRA*^13^. This weak association may be due to the relatively small sample size, which might have reduced the statistical power of the analysis. In contrast to corneal astigmatism, the *PDGFRA* region has not been associated with refractive astigmatism: Lopes et al. reported no association between refractive astigmatism and *PDGFRA* in a GWAS meta-analysis with European ancestry cohorts (n = 22,100)^33^. Likewise, a larger GWAS meta-analysis with European and Asian ancestry cohorts (n = 45,931) reported lack of association between refractive astigmatism and *PDGFRA*^34^. In addition, another large-scale GWAS for refractive astigmatism analyzed as a continuous trait also reported no association between refractive astigmatism and *PDGFRA* in a European ancestry cohort (n = 88,005)^12^. The nonexistence of these associations may be due to the facts that the trait of refractive astigmatism, which consists of corneal and internal astigmatism, is different from that of corneal astigmatism, indicating a difference in genetic background between corneal astigmatism and internal astigmatism that arises from the internal optics of the eye (mainly the crystalline lens).

One of the limitations of the present study is the smaller sample size compared with previous studies. This limitation possibly lad to reduced statistical power and increased the risk of type II error. The statistical power of this study to replicate the association between corneal astigmatism and rs7677751 (OR = 1.26) originally reported by Fan et al.^9^ ranged from 35.0% to 51.8% in case–control analyses with the stratified patient groups (≤ − 0.75 D: 51.8%; ≤  − 1.00 D: 47.6%; ≤  − 1.25 D: 41.9%; ≤  − 1.50 D: 35.0%). Thus, the present study is underpowered to detect the genetic associations described in the previous studies and may have led to the failure in detecting some true associations in a Japanese population. To overcome this issue and validate our findings, further genetic studies with larger sample sizes from a Japanese population are needed. Another limitation of the present study is that the functional role of the identified SNPs could not be clarified. Although publicly available data suggest that they may affect the transcriptional regulation of *PDGFRA*, no concordant results were observed throughout the database analysis. Thus, further studies are needed to elucidate the functional implications of the identified *PDGFRA* SNPs.

In conclusion, to our knowledge, this study is the first to investigate the association between the *PDGFRA* gene region and corneal astigmatism in individuals of Japanese ancestry. We found that SNPs in the upstream region of *PDGFRA* were associated with higher corneal astigmatism in our Japanese population, with ORs increasing with increasing degree of corneal astigmatism. Our findings suggest the possibility that these *PDGFRA* SNPs are potential genetic factors in the development of greater corneal astigmatism. To clarify the contribution of *PDGFRA* SNPs to the risk of corneal astigmatism, further genetic studies using other ethnic populations and that take into account the degree of corneal astigmatism are needed.

## Methods

### Participants

A total of 2377 unrelated Japanese individuals were recruited from Yokohama City University, Okada Eye Clinic, and Aoto Eye Clinic in Yokohama, Kanagawa Prefecture, Japan. Corneal curvature radii were measured in horizontal and vertical meridians with autorefractors (ARK-730A, [NIDEK, Aichi, Japan], ARK-700A [NIDEK], and KP-8100P [TOPCON, Tokyo, Japan]). The keratometric index of 1.3375 was used to convert the radius of corneal curvature in millimeters into a corneal power in diopters (D). Corneal cylinder power was calculated as the difference in diopters between the steepest and flattest medians. According to previous studies^9,11^, we defined individuals with mean corneal cylinder power ≤  − 0.75 D across both eyes as corneal astigmatism cases (n = 1535), while controls were defined as mean corneal cylinder power >  − 0.75 D across both eyes (n = 842). This study did not include individuals who had a previous history of ocular surgery or any ocular condition that may affect the accuracy of keratometry. The study details were explained to all participants, and written informed consent was obtained from all participants. The study methodology adhered to the tenets of the Declaration of Helsinki and was approved by the Ethics Committee of Yokohama City University School of Medicine (approval number: A150122004; approval date: 06/02/2015).

### SNP genotyping

Genomic DNA was extracted from peripheral blood samples with the QIAamp DNA Blood Maxi Kit (Qiagen, Hilden, Germany). Procedures were performed under standardized conditions to prevent variation in DNA quality.

We selected nine tagging SNPs (rs11133315, rs6554162, rs2303429, rs7656613, rs1547905, rs3816888, rs17739921, rs1826426, and rs6554170) that together covered the entire *PDGFRA* gene region, including 15 kb upstream and downstream, from HapMap Japanese data (minor allele frequency ≥ 1%, pairwise *r*^2^ ≥ 0.8), with the LD TAG SNP selection tool in the SNPinfo web server (https://snpinfo.niehs.nih.gov/). We evaluated four SNPs reportedly associated with corneal astigmatism in previous studies (rs7673984, rs4864857, rs6554163, and rs7677751)^9–12^. Genotyping was performed with the TaqMan 5´ exonuclease assay and primer–probe sets supplied by Thermo Fisher Scientific Inc. (Foster City, CA, USA). The polymerase chain reaction (PCR) for each SNP was performed with a 10-μL reaction mixture that contained 1 × TaqMan GTXpress Master Mix (Thermo Fisher Scientific), 1 × TaqMan SNP Genotyping Assay primer/probe mix, and 3 ng of genomic DNA. The PCR conditions were as follows: 95 °C for 20 s, followed by 40 cycles of denaturation at 95 °C for 3 s and annealing/extension at 60 °C for 20 s. The fluorescent probe signal was detected with the StepOnePlus Real-Time PCR System (Thermo Fisher Scientific), according to the manufacturer’s instructions.

### Imputation analysis

We performed imputation analysis using the MACH v1.0 program (http://www.sph.umich.edu/csg/abecasis/MACH/index.html)^35,36^ to evaluate potential associations with un-genotyped SNPs in the *PDGFRA* gene region. As a reference panel, we used the 1000 Genomes Phase 3 datasets of 504 East Asian samples, which included a set of Japanese samples from Tokyo (JPT, n = 104), Han Chinese samples from Beijing (CHB, n = 103), Southern Han Chinese samples (CHS, n = 105), Chinese Dai samples from Xishuangbanna (CDX, n = 93), and Kinh samples from Ho Chi Minh City (KHV, n = 99) (http://www.1000genomes.org/)^37^. All imputed SNPs were filtered with the following quality control parameters: HWE *P* > 0.05, minor allele frequency > 0.01, and squared correlation between imputed and true genotypes (Rsq) > 0.7. After the quality control filtering, we included the 179 imputed SNPs in further analysis.

To highlight the potentially causal SNP in the *PDGFRA* gene region, we performed fine-mapping with FINEMAP v1.2 software^14^. This software uses a shotgun stochastic search algorithm. We ran FINEMAP with the assumption that there would be only one causal variant in the gene region. We calculated the posterior inclusion probabilities and log_10_ Bayes factor to assess the causality of each SNP within the *PDGFRA* gene region.

### Statistical analysis

Association analyses were carried out under an additive model using SNP & Variation Suite software version 8.8.3 (Golden Helix, Inc., Bozeman, MT, USA). Age and sex were included in the model as covariates. The obtained *P*-values were corrected for multiple testing with the Bonferroni correction based on the number of tagging SNPs tested (n = 9), because one of the tagging SNPs, rs11133315, was in strong LD (*r*^2^ ≥ 0.87) with four SNPs reportedly associated with corneal astigmatism (rs7673984, rs4864857, rs6554163, and rs7677751). A *P*c-value < 0.05 was considered significant. We generated a regional association plot for the *PDGFRA* gene region using LocusZoom (http://csg.sph.umich.edu/locuszoom/)^38^. LD between SNPs was assessed using Haploview 4.2 software^39^ and LocusZoom. Statistical power calculations were performed using Sampsize calculator (http://sampsize.sourceforge.net/).

### Functional annotation

We investigated the functional roles of the identified SNPs using HaploReg v4.2 (https://pubs.broadinstitute.org/mammals/haploreg/haploreg.php) and RegulomeDB (https://www.regulomedb.org/)^15,16^. We also used the GTEx Portal online database, version 8 (https://www.gtexportal.org/home/) to evaluate the eQTL effect of the identified SNPs^17^.

### Supplementary Information

Supplementary Information.

## Data Availability

All data generated or analyzed during this study are included in this published article.
