# Supplementary material for: Association of PDGFRA polymorphisms with the risk of corneal astigmatism in a Japanese population
Source: Sci Rep. 2023 Sep 26;13:16075. doi: 10.1038/s41598-023-43333-1 (PMC10522672; doi:10.1038/s41598-023-43333-1)
Supplement: Supplementary file 1 — Supplementary Information. [file 41598_2023_43333_MOESM1_ESM.pdf]

SUPPLEMENTARY INFORMATION

Association of *PDGFRA* polymorphisms with the risk of corneal astigmatism in a Japanese population

Hideharu Fukasaku[1,2], Akira Meguro[3,4*], Masaki Takeuchi[3,4], Nobuhisa Mizuki[3,4], Masao Ota[4,5], Kengo Funakoshi[1]

[1]Department of Neuroanatomy, Yokohama City University Graduate School of Medicine, Yokohama, Kanagawa 236-0004, Japan
[2]Fukasaku Eye Institute, Yokohama, Kanagawa 220-0003, Japan
[3]Department of Ophthalmology and Visual Science, Yokohama City University Graduate School of Medicine, Yokohama, Kanagawa 236-0004, Japan
[4]Department of Advanced Medicine for Ocular Diseases, Yokohama City University Graduate School of Medicine, Yokohama, Kanagawa 236-0004, Japan
[5]Department of Medicine, Division of Hepatology and Gastroenterology, Shinshu University School of Medicine, Matsumoto, Nagano 390-8621, Japan
[*]Corresponding author: akmeguro@yokohama-cu.ac.jp

CONTENTS

Supplementary Figure

Supplementary Figure S1. Linkage disequilibrium plot of 13 genotyped SNPs in the *PDGFRA* gene region.

Supplementary Tables

Supplementary Table S1. Association and fine mapping results for 192 SNPs in the *PDGFRA* gene region in cases with corneal astigmatism ≤−1.25 D.

Supplementary Table S2. Association and fine mapping results for 192 SNPs in the *PDGFRA* gene region in cases with corneal astigmatism ≤−1.50 D.

Supplementary Table S3. Association and fine mapping results for 192 SNPs in the *PDGFRA* gene region in cases with corneal astigmatism ≤−0.75 D.

Supplementary Table S4. Association and fine mapping results for 192 SNPs in the *PDGFRA* gene region in cases with corneal astigmatism ≤−1.00 D.

Supplementary Table S5. Functional annotation of the lead SNPs in the *PDGFRA* gene region identified in this study.

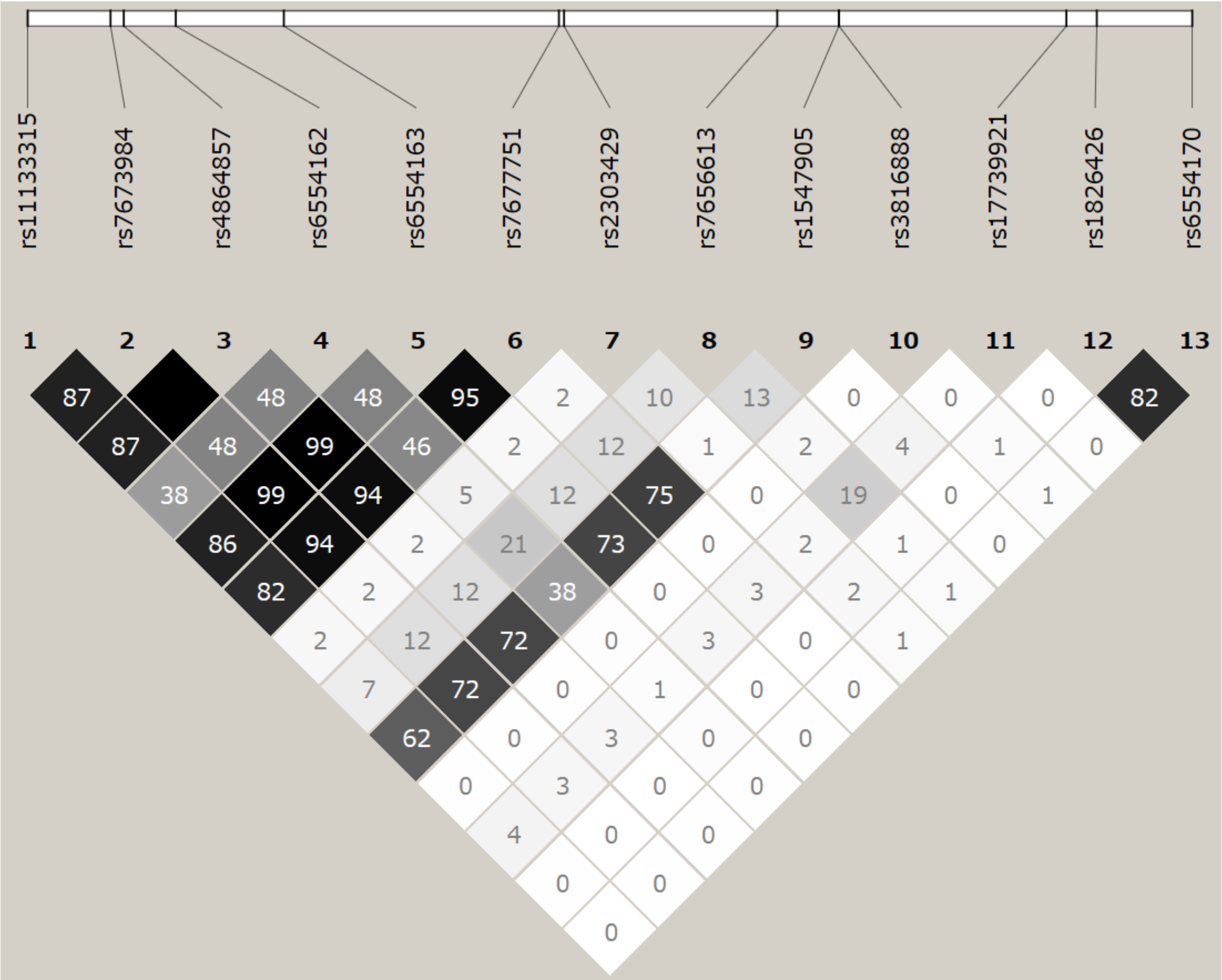

Supplementary Figure S1. Linkage disequilibrium plot of 13 genotyped SNPs in the *PDGFRA* gene region.

For each SNP pair, the corresponding r^2 value is shown as a percentage within the respective square. Higher r^2 values are indicated by darker shading of the square's background.

Supplementary Table S1. Association and fine mapping results for 192 SNPs in the *PDGFRA* gene region in the cases with corneal astigmatism ≤ − 1.25 D

SNP	Position on Chr. 4 (GRCh37)	Alleles (1>2)	Minor Allele Freq.		*P*	*P* c	OR (95% CI)	Fine Mapping	
			Cases (N=787)	Controls (N=842)				PIP	$Log_{10}BF$
rs17084040	55,074,368	G>A	0.128	0.127	0.80		1.03 (0.82-1.30)	0.00033	-1.20
rs17084042	55,074,542	G>T	0.128	0.127	0.80		1.03 (0.82-1.30)	0.00033	-1.20
rs57217483	55,074,922	G>T	0.128	0.127	0.80		1.03 (0.82-1.30)	0.00033	-1.20
rs28431840	55,075,792	T>G	0.128	0.127	0.80		1.03 (0.82-1.30)	0.00033	-1.20
rs6832597	55,076,237	C>A	0.220	0.178	0.0028	0.026	1.34 (1.11-1.63)	0.011	0.33
rs6831380	55,076,345	G>A	0.220	0.178	0.0028	0.026	1.34 (1.11-1.63)	0.011	0.33
rs7676972	55,077,078	C>T	0.220	0.178	0.0028	0.026	1.34 (1.11-1.63)	0.011	0.33
rs77086742	55,077,348	A>G	0.128	0.127	0.80		1.03 (0.82-1.30)	0.00033	-1.20
rs6554160	55,077,549	T>A	0.220	0.178	0.0028	0.026	1.34 (1.11-1.63)	0.011	0.33
rs111944671	55,078,108	G>A	0.227	0.200	0.029	0.26	1.23 (1.02-1.49)	0.0022	-0.38
rs28436914	55,078,194	A>T	0.227	0.200	0.029	0.26	1.23 (1.02-1.49)	0.0022	-0.38
rs28753397	55,078,496	C>T	0.227	0.200	0.029	0.26	1.23 (1.02-1.49)	0.0022	-0.38
rs139145626	55,078,614	C>T	0.220	0.178	0.0028	0.026	1.34 (1.11-1.63)	0.011	0.33
rs718454	55,079,544	G>C	0.227	0.200	0.029	0.26	1.23 (1.02-1.49)	0.0022	-0.38
rs718455	55,079,614	A>C	0.227	0.200	0.029	0.26	1.23 (1.02-1.49)	0.0022	-0.38
rs12648347	55,080,051	A>G	0.227	0.200	0.029	0.26	1.23 (1.02-1.49)	0.0022	-0.38
rs12505054	55,080,371	T>G	0.227	0.200	0.029	0.26	1.23 (1.02-1.49)	0.0022	-0.38
rs7673267	55,081,133	C>T	0.227	0.200	0.029	0.26	1.23 (1.02-1.49)	0.0022	-0.38
rs11133312	55,081,744	C>T	0.227	0.200	0.029	0.26	1.23 (1.02-1.49)	0.0022	-0.38
rs11133313	55,081,757	C>T	0.227	0.200	0.029	0.26	1.23 (1.02-1.49)	0.0022	-0.38
rs11133314	55,081,850	C>A	0.227	0.200	0.029	0.26	1.23 (1.02-1.49)	0.0022	-0.38
rs11133315	55,082,158	G>A	0.220	0.178	0.0028	0.026	1.34 (1.11-1.63)	0.011	0.33
rs4398585	55,082,353	C>T	0.227	0.200	0.029	0.26	1.23 (1.02-1.49)	0.0022	-0.38
rs28441665	55,083,056	T>C	0.128	0.127	0.80		1.03 (0.82-1.30)	0.00033	-1.20
rs4864855	55,083,451	G>T	0.227	0.200	0.029	0.26	1.23 (1.02-1.49)	0.0022	-0.38
rs4864856	55,084,720	G>A	0.227	0.200	0.029	0.26	1.23 (1.02-1.49)	0.0022	-0.38
rs7683707	55,086,036	T>C	0.227	0.200	0.029	0.26	1.23 (1.02-1.49)	0.0022	-0.38
rs60806423	55,086,816	C>T	0.199	0.156	0.0018	0.016	1.38 (1.13-1.69)	0.016	0.48
rs192263827	55,087,113	T>C	0.102	0.111	0.062		0.79 (0.62-1.01)	0.0013	-0.61
rs113828936	55,087,144	G>A	0.199	0.156	0.0018	0.016	1.38 (1.13-1.69)	0.016	0.48
rs17084051	55,087,581	C>A	0.199	0.156	0.0018	0.016	1.38 (1.13-1.69)	0.016	0.48
rs28622224	55,088,093	C>T	0.328	0.283	0.0056	0.0502	1.27 (1.07-1.50)	0.0069	0.12
rs61320297	55,088,175	G>A	0.199	0.156	0.0018	0.016	1.38 (1.13-1.69)	0.016	0.48
rs7673597	55,088,543	C>T	0.199	0.156	0.0018	0.016	1.38 (1.13-1.69)	0.016	0.48
rs7673625	55,088,586	C>T	0.199	0.156	0.0018	0.016	1.38 (1.13-1.69)	0.016	0.48
rs7673984	55,088,761	C>T	0.199	0.156	0.0018	0.016	1.38 (1.13-1.69)	0.016	0.48
rs4394060	55,088,909	C>A	0.199	0.156	0.0018	0.016	1.38 (1.13-1.69)	0.016	0.48
rs4864857	55,089,814	T>C	0.199	0.156	0.0018	0.016	1.38 (1.13-1.69)	0.016	0.48
rs4864858	55,089,953	T>A	0.199	0.156	0.0018	0.016	1.38 (1.13-1.69)	0.016	0.48
rs4864859	55,090,015	A>G	0.199	0.156	0.0018	0.016	1.38 (1.13-1.69)	0.016	0.48
rs4864860	55,090,021	T>C	0.199	0.156	0.0018	0.016	1.38 (1.13-1.69)	0.016	0.48
rs60863210	55,090,099	T>C	0.199	0.156	0.0018	0.016	1.38 (1.13-1.69)	0.016	0.48
rs7698425	55,090,656	C>T	0.199	0.156	0.0018	0.016	1.38 (1.13-1.69)	0.016	0.48
rs7681399	55,090,886	T>G	0.199	0.156	0.0018	0.016	1.38 (1.13-1.69)	0.016	0.48
rs2114039	55,092,626	T>C	0.328	0.284	0.0072	0.065	1.26 (1.06-1.49)	0.0057	0.04
rs1800809	55,093,914	A>G	0.199	0.157	0.0025	0.022	1.37 (1.12-1.67)	0.012	0.38
rs6554162	55,093,955	G>A	0.328	0.284	0.0072	0.065	1.26 (1.06-1.49)	0.0057	0.04
rs1800810	55,094,031	C>G	0.199	0.157	0.0025	0.022	1.37 (1.12-1.67)	0.012	0.38
rs1800813	55,094,467	G>A	0.199	0.157	0.0025	0.022	1.37 (1.12-1.67)	0.012	0.38
rs1800812	55,094,629	G>T	0.199	0.157	0.0025	0.022	1.37 (1.12-1.67)	0.012	0.38
rs7690503	55,096,150	C>G	0.199	0.157	0.0025	0.022	1.37 (1.12-1.67)	0.012	0.38
rs7673027	55,096,180	T>A	0.199	0.157	0.0025	0.022	1.37 (1.12-1.67)	0.012	0.38
rs7668190	55,096,270	A>T	0.328	0.284	0.0072	0.065	1.26 (1.06-1.49)	0.0057	0.04
rs7689569	55,096,398	G>A	0.199	0.157	0.0025	0.022	1.37 (1.12-1.67)	0.012	0.38
rs7673853	55,096,606	T>C	0.199	0.157	0.0025	0.022	1.37 (1.12-1.67)	0.012	0.38
rs7679903	55,097,373	T>C	0.199	0.157	0.0025	0.022	1.37 (1.12-1.67)	0.012	0.38
rs890203	55,097,405	A>C	0.199	0.157	0.0025	0.022	1.37 (1.12-1.67)	0.012	0.38
rs4864861	55,097,685	C>T	0.199	0.157	0.0025	0.022	1.37 (1.12-1.67)	0.012	0.38
rs4864504	55,097,835	G>C	0.328	0.284	0.0072	0.065	1.26 (1.06-1.49)	0.0057	0.04
rs4608869	55,098,936	G>C	0.199	0.157	0.0025	0.022	1.37 (1.12-1.67)	0.012	0.38
rs4368668	55,098,954	A>G	0.199	0.157	0.0025	0.022	1.37 (1.12-1.67)	0.012	0.38
rs4635872	55,099,041	C>A	0.199	0.157	0.0025	0.022	1.37 (1.12-1.67)	0.012	0.38
rs6850748	55,099,164	T>G	0.199	0.157	0.0025	0.022	1.37 (1.12-1.67)	0.012	0.38
rs4864862	55,100,489	G>A	0.199	0.157	0.0025	0.022	1.37 (1.12-1.67)	0.012	0.38
rs4864863	55,100,831	A>G	0.199	0.157	0.0025	0.022	1.37 (1.12-1.67)	0.012	0.38
rs7678144	55,102,425	T>C	0.199	0.157	0.0025	0.022	1.37 (1.12-1.67)	0.012	0.38

Supplementary Table S1. (Cont.)

SNP	Position on Chr. 4 (GRCh37)	Alleles (1>2)	Minor Allele Freq. Cases (N=787)	Minor Allele Freq. Controls (N=842)	*P*	*P*c	OR (95% CI)	Fine Mapping PIP	Fine Mapping $Log_{10}BF$
rs6554163	55,102,559	T>A	0.199	0.157	0.0025	0.022	1.37 (1.12-1.67)	0.012	0.38
rs6836215	55,102,741	T>C	0.199	0.157	0.0025	0.022	1.37 (1.12-1.67)	0.012	0.38
rs67432867	55,104,454	A>T	0.199	0.157	0.0025	0.022	1.37 (1.12-1.67)	0.012	0.38
rs73252942	55,104,604	T>C	0.199	0.157	0.0025	0.022	1.37 (1.12-1.67)	0.012	0.38
rs6554164	55,106,695	T>C	0.328	0.283	0.0068	0.061	1.26 (1.07-1.49)	0.0060	0.06
rs35714192	55,107,750	G>C	0.470	0.437	0.22		1.10 (0.94-1.29)	0.00060	-0.94
rs147558377	55,113,623	C>G	0.198	0.156	0.0023	0.020	1.37 (1.12-1.68)	0.013	0.41
rs13435164	55,115,828	C>G	0.327	0.283	0.0070	0.063	1.26 (1.06-1.49)	0.0059	0.05
rs6554165	55,115,847	G>A	0.327	0.283	0.0070	0.063	1.26 (1.06-1.49)	0.0059	0.05
rs79938737	55,116,048	G>C	0.128	0.126	0.79		1.03 (0.82-1.30)	0.00033	-1.20
rs60251664	55,117,805	T>C	0.128	0.126	0.79		1.03 (0.82-1.30)	0.00033	-1.20
rs76091088	55,119,631	T>C	0.102	0.111	0.062		0.79 (0.62-1.01)	0.0013	-0.61
rs4422471	55,121,921	C>T	0.197	0.154	0.0022	0.019	1.37 (1.12-1.68)	0.014	0.42
rs10029499	55,122,494	A>G	0.326	0.281	0.0060	0.054	1.27 (1.07-1.50)	0.0065	0.10
rs7677751	55,124,460	C>T	0.192	0.150	0.0039	0.035	1.35 (1.10-1.66)	0.0089	0.23
rs9991165	55,124,591	A>G	0.187	0.146	0.0037	0.033	1.36 (1.10-1.67)	0.0092	0.25
rs2303429	55,124,870	C>T	0.102	0.111	0.062		0.79 (0.62-1.01)	0.0013	-0.61
rs2303430	55,125,058	A>T	0.453	0.471	0.77		1.02 (0.88-1.19)	0.00033	-1.19
rs73252946	55,125,992	G>A	0.185	0.146	0.0060	0.054	1.33 (1.09-1.64)	0.0065	0.10
rs58435984	55,127,990	T>C	0.185	0.146	0.0060	0.054	1.33 (1.09-1.64)	0.0065	0.10
rs2229307	55,130,078	T>C	0.185	0.146	0.0060	0.054	1.33 (1.09-1.64)	0.0065	0.10
rs2307049	55,130,154	G>A	0.185	0.146	0.0060	0.054	1.33 (1.09-1.64)	0.0065	0.10
rs67279506	55,130,682	A>G	0.185	0.146	0.0060	0.054	1.33 (1.09-1.64)	0.0065	0.10
rs58727676	55,130,794	T>G	0.185	0.146	0.0060	0.054	1.33 (1.09-1.64)	0.0065	0.10
rs7688997	55,131,450	C>A	0.185	0.146	0.0060	0.054	1.33 (1.09-1.64)	0.0065	0.10
rs56145315	55,131,689	C>T	0.185	0.146	0.0060	0.054	1.33 (1.09-1.64)	0.0065	0.10
rs28600756	55,131,732	C>A	0.185	0.146	0.0060	0.054	1.33 (1.09-1.64)	0.0065	0.10
rs67600360	55,132,162	A>G	0.185	0.146	0.0060	0.054	1.33 (1.09-1.64)	0.0065	0.10
rs4864864	55,132,325	C>T	0.185	0.146	0.0060	0.054	1.33 (1.09-1.64)	0.0065	0.10
rs4864865	55,132,878	T>A	0.185	0.146	0.0060	0.054	1.33 (1.09-1.64)	0.0065	0.10
rs73252948	55,133,252	T>C	0.185	0.146	0.0060	0.054	1.33 (1.09-1.64)	0.0065	0.10
rs4358459	55,133,726	T>G	0.185	0.146	0.0060	0.054	1.33 (1.09-1.64)	0.0065	0.10
rs28489067	55,133,936	C>T	0.185	0.146	0.0060	0.054	1.33 (1.09-1.64)	0.0065	0.10
rs28650939	55,133,959	C>T	0.185	0.146	0.0060	0.054	1.33 (1.09-1.64)	0.0065	0.10
rs28528897	55,134,339	C>G	0.185	0.146	0.0060	0.054	1.33 (1.09-1.64)	0.0065	0.10
rs7660560	55,134,394	G>A	0.185	0.146	0.0060	0.054	1.33 (1.09-1.64)	0.0065	0.10
rs7691129	55,134,466	T>C	0.185	0.146	0.0060	0.054	1.33 (1.09-1.64)	0.0065	0.10
rs7686588	55,134,628	A>G	0.185	0.146	0.0060	0.054	1.33 (1.09-1.64)	0.0065	0.10
rs12641563	55,135,447	C>G	0.185	0.146	0.0060	0.054	1.33 (1.09-1.64)	0.0065	0.10
rs12644709	55,135,580	A>G	0.185	0.146	0.0060	0.054	1.33 (1.09-1.64)	0.0065	0.10
rs12644749	55,135,771	A>G	0.185	0.146	0.0060	0.054	1.33 (1.09-1.64)	0.0065	0.10
rs12508225	55,136,027	G>C	0.185	0.146	0.0060	0.054	1.33 (1.09-1.64)	0.0065	0.10
rs12506290	55,136,132	T>A	0.185	0.146	0.0060	0.054	1.33 (1.09-1.64)	0.0065	0.10
rs12505491	55,136,245	A>G	0.185	0.146	0.0060	0.054	1.33 (1.09-1.64)	0.0065	0.10
rs1565669	55,137,183	T>C	0.185	0.146	0.0060	0.054	1.33 (1.09-1.64)	0.0065	0.10
rs1565670	55,137,281	A>G	0.185	0.146	0.0060	0.054	1.33 (1.09-1.64)	0.0065	0.10
rs35597368	55,139,771	T>C	0.172	0.139	0.018	0.17	1.29 (1.04-1.59)	0.0030	-0.25
rs869978	55,140,016	C>T	0.288	0.256	0.032	0.29	1.21 (1.02-1.44)	0.0020	-0.41
rs2307050	55,141,293	G>A	0.172	0.139	0.018	0.17	1.29 (1.04-1.59)	0.0030	-0.25
rs73252950	55,141,500	C>T	0.172	0.139	0.017	0.16	1.29 (1.05-1.59)	0.0031	-0.23
rs6855761	55,141,532	A>T	0.172	0.139	0.017	0.16	1.29 (1.05-1.59)	0.0031	-0.23
rs7656613	55,141,843	T>C	0.449	0.464	0.61		1.04 (0.89-1.21)	0.00036	-1.17
rs1105825	55,142,205	G>A	0.170	0.138	0.024	0.22	1.27 (1.03-1.57)	0.0025	-0.33
rs67388297	55,142,691	C>G	0.170	0.138	0.023	0.21	1.28 (1.03-1.58)	0.0025	-0.31
rs28698464	55,143,110	A>G	0.170	0.137	0.021	0.19	1.28 (1.04-1.58)	0.0027	-0.29
rs1316926	55,143,286	G>A	0.245	0.258	0.23		0.90 (0.75-1.07)	0.00058	-0.96
rs28374326	55,143,321	C>T	0.172	0.138	0.018	0.17	1.29 (1.04-1.59)	0.0030	-0.25
rs10028020	55,143,577	G>A	0.173	0.138	0.019	0.17	1.29 (1.04-1.59)	0.0029	-0.25
rs1907819	55,144,420	G>A	0.179	0.145	0.030	0.27	1.26 (1.02-1.55)	0.0021	-0.39
rs2412556	55,145,258	A>G	0.179	0.145	0.033	0.30	1.25 (1.02-1.54)	0.0020	-0.42
rs4289498	55,145,432	A>G	0.179	0.145	0.033	0.30	1.25 (1.02-1.54)	0.0020	-0.42
rs1547904	55,146,389	C>T	0.179	0.145	0.033	0.30	1.25 (1.02-1.54)	0.0020	-0.42
rs1547905	55,146,754	C>A	0.179	0.145	0.033	0.30	1.25 (1.02-1.54)	0.0020	-0.42
rs3816888	55,146,761	C>T	0.020	0.021	0.54		1.18 (0.69-2.03)	0.00037	-1.15
rs58025349	55,146,927	A>T	0.179	0.145	0.033	0.30	1.25 (1.02-1.54)	0.0020	-0.42
rs2291591	55,147,769	C>T	0.088	0.093	0.76		0.96 (0.73-1.26)	0.00034	-1.19
rs28811736	55,148,547	C>G	0.179	0.145	0.033	0.30	1.25 (1.02-1.54)	0.0020	-0.42

Supplementary Table S1. (Cont.)

SNP	Position on Chr. 4 (GRCh37)	Alleles (1>2)	Minor Allele Freq.		P	Pc	OR (95% CI)	Fine Mapping	
			Cases (N=787)	Controls (N=842)				PIP	$Log_{10}BF$
rs7677708	55,149,258	A>G	0.179	0.145	0.033	0.30	1.25 (1.02-1.54)	0.0020	-0.42
rs2412557	55,149,457	A>C	0.179	0.145	0.033	0.30	1.25 (1.02-1.54)	0.0020	-0.42
rs2412558	55,149,507	A>G	0.179	0.145	0.033	0.30	1.25 (1.02-1.54)	0.0020	-0.42
rs2228230	55,152,040	C>T	0.175	0.144	0.047	0.43	1.24 (1.00-1.52)	0.0016	-0.53
rs4864872	55,152,284	G>T	0.175	0.144	0.047	0.43	1.24 (1.00-1.52)	0.0016	-0.53
rs11940889	55,152,583	C>T	0.175	0.144	0.047	0.43	1.24 (1.00-1.52)	0.0016	-0.53
rs11931555	55,152,715	G>C	0.176	0.144	0.039	0.35	1.25 (1.01-1.54)	0.0018	-0.47
rs10020847	55,153,134	C>T	0.175	0.144	0.047	0.43	1.24 (1.00-1.52)	0.0016	-0.53
rs144143098	55,153,551	A>G	0.020	0.021	0.92		1.03 (0.60-1.75)	0.00032	-1.21
rs10021728	55,154,109	C>T	0.175	0.144	0.047	0.43	1.24 (1.00-1.52)	0.0016	-0.53
rs9993187	55,154,250	T>C	0.175	0.144	0.047	0.43	1.24 (1.00-1.52)	0.0016	-0.53
rs11733839	55,154,527	G>C	0.175	0.144	0.047	0.43	1.24 (1.00-1.52)	0.0016	-0.53
rs55732997	55,154,891	T>C	0.175	0.144	0.047	0.43	1.24 (1.00-1.52)	0.0016	-0.53
rs10004857	55,155,980	G>A	0.175	0.144	0.047	0.43	1.24 (1.00-1.52)	0.0016	-0.53
rs11133317	55,156,091	G>T	0.175	0.144	0.047	0.43	1.24 (1.00-1.52)	0.0016	-0.53
rs4864875	55,156,166	A>G	0.175	0.141	0.026	0.24	1.27 (1.03-1.56)	0.0023	-0.35
rs4864876	55,156,300	C>T	0.175	0.141	0.026	0.24	1.27 (1.03-1.56)	0.0023	-0.35
rs2276948	55,156,400	G>A	0.175	0.141	0.026	0.24	1.27 (1.03-1.56)	0.0023	-0.35
rs13147194	55,157,134	T>G	0.175	0.141	0.026	0.24	1.27 (1.03-1.56)	0.0023	-0.35
rs10010509	55,157,206	G>T	0.175	0.141	0.026	0.24	1.27 (1.03-1.56)	0.0023	-0.35
rs10003055	55,157,666	T>G	0.174	0.141	0.028	0.25	1.27 (1.03-1.56)	0.0022	-0.37
rs11935157	55,158,514	A>T	0.173	0.140	0.030	0.27	1.26 (1.02-1.56)	0.0021	-0.39
rs139471049	55,158,597	A>G	0.086	0.090	0.86		0.98 (0.74-1.28)	0.00033	-1.20
rs60218083	55,158,938	A>G	0.171	0.139	0.035	0.31	1.26 (1.02-1.55)	0.0019	-0.43
rs55784333	55,159,391	C>G	0.171	0.139	0.035	0.31	1.26 (1.02-1.55)	0.0019	-0.43
rs10032688	55,160,658	G>A	0.171	0.139	0.035	0.31	1.26 (1.02-1.55)	0.0019	-0.43
rs3733540	55,161,254	T>C	0.171	0.139	0.035	0.31	1.26 (1.02-1.55)	0.0019	-0.43
rs3690	55,161,813	A>C	0.171	0.139	0.035	0.31	1.26 (1.02-1.55)	0.0019	-0.43
rs17739921	55,164,866	A>C	0.274	0.285	0.22		0.90 (0.76-1.06)	0.00059	-0.94
rs34491905	55,165,075	C>T	0.274	0.283	0.25		0.91 (0.77-1.07)	0.00054	-0.98
rs1961871	55,167,266	G>A	0.352	0.357	0.59		1.04 (0.89-1.22)	0.00036	-1.16
rs1826426	55,167,287	A>G	0.402	0.403	0.31		1.08 (0.93-1.27)	0.00049	-1.03
rs10000267	55,167,931	A>G	0.372	0.374	0.44		1.06 (0.91-1.24)	0.00041	-1.11
rs11722786	55,169,182	C>T	0.352	0.357	0.59		1.04 (0.89-1.22)	0.00036	-1.16
rs11727002	55,169,183	A>G	0.352	0.357	0.59		1.04 (0.89-1.22)	0.00036	-1.16
rs55836714	55,170,352	T>C	0.371	0.374	0.45		1.06 (0.91-1.24)	0.00041	-1.11
rs77890222	55,170,462	C>A	0.352	0.357	0.59		1.04 (0.89-1.22)	0.00036	-1.16
rs62299407	55,171,129	A>G	0.352	0.357	0.59		1.04 (0.89-1.22)	0.00036	-1.16
rs4594778	55,172,289	C>T	0.352	0.357	0.59		1.04 (0.89-1.22)	0.00036	-1.16
rs4864877	55,172,357	A>G	0.352	0.357	0.59		1.04 (0.89-1.22)	0.00036	-1.16
rs13353653	55,172,713	G>A	0.352	0.357	0.59		1.04 (0.89-1.22)	0.00036	-1.16
rs4864878	55,173,715	C>T	0.352	0.357	0.59		1.04 (0.89-1.22)	0.00036	-1.16
rs6858442	55,173,933	G>A	0.352	0.357	0.59		1.04 (0.89-1.22)	0.00036	-1.16
rs6554168	55,174,827	G>A	0.352	0.357	0.59		1.04 (0.89-1.22)	0.00036	-1.16
rs6554170	55,174,885	C>T	0.352	0.357	0.59		1.04 (0.89-1.22)	0.00036	-1.16
rs1565668	55,175,601	T>C	0.352	0.357	0.59		1.04 (0.89-1.22)	0.00036	-1.16
rs1565665	55,175,768	C>G	0.352	0.357	0.59		1.04 (0.89-1.22)	0.00036	-1.16
rs28889275	55,176,073	G>A	0.352	0.357	0.59		1.04 (0.89-1.22)	0.00036	-1.16
rs28884174	55,176,383	T>C	0.352	0.357	0.59		1.04 (0.89-1.22)	0.00036	-1.16
rs28707743	55,178,018	C>T	0.350	0.356	0.62		1.04 (0.89-1.22)	0.00036	-1.17
rs28547147	55,179,059	C>A	0.351	0.357	0.61		1.04 (0.89-1.22)	0.00036	-1.16
rs10016845	55,183,077	T>C	0.350	0.356	0.62		1.04 (0.89-1.22)	0.00036	-1.17
rs11732117	55,184,119	A>G	0.350	0.356	0.62		1.04 (0.89-1.22)	0.00036	-1.17
rs6850695	55,185,035	C>T	0.350	0.356	0.62		1.04 (0.89-1.22)	0.00036	-1.17
rs10028483	55,186,168	T>C	0.350	0.356	0.62		1.04 (0.89-1.22)	0.00036	-1.17
rs9991681	55,186,390	G>A	0.349	0.356	0.64		1.04 (0.89-1.22)	0.00035	-1.17
rs28714526	55,186,508	A>G	0.350	0.356	0.62		1.04 (0.89-1.22)	0.00036	-1.17
rs2087806	55,186,956	C>T	0.350	0.356	0.62		1.04 (0.89-1.22)	0.00036	-1.17
rs10029431	55,187,406	A>T	0.349	0.356	0.64		1.04 (0.89-1.22)	0.00035	-1.17

SNP, single-nucleotide polymorphism; 1, major allele; 2, minor allele; Pc, corrected P-value; OR, odds ratio; CI, confidence interval; PIP, posterior inclusion probability; $Log_{10}BF$, log_{10} Bayes factor.

Supplementary Table S2. Association and fine mapping results for 192 SNPs in the *PDGFRA* gene region in the cases with corneal astigmatism ≤ −1.50 D

SNP	Position on Chr. 4 (GRCh37)	Alleles (1>2)	Minor Allele Freq. Cases (N=514)	Minor Allele Freq. Controls (N=842)	*P*	*P*c	OR (95% CI)	Fine Mapping PIP	Fine Mapping $Log_{10}BF$
rs17084040	55,074,368	G>A	0.132	0.127	0.61		1.07 (0.82-1.40)	0.00071	-0.87
rs17084042	55,074,542	G>T	0.132	0.127	0.61		1.07 (0.82-1.40)	0.00071	-0.87
rs57217483	55,074,922	G>T	0.132	0.127	0.61		1.07 (0.82-1.40)	0.00071	-0.87
rs28431840	55,075,792	T>G	0.132	0.127	0.61		1.07 (0.82-1.40)	0.00071	-0.87
rs6832597	55,076,237	C>A	0.217	0.178	0.0043	0.038	1.39 (1.11-1.73)	0.015	0.46
rs6831380	55,076,345	G>A	0.217	0.178	0.0043	0.038	1.39 (1.11-1.73)	0.015	0.46
rs7676972	55,077,078	C>T	0.217	0.178	0.0043	0.038	1.39 (1.11-1.73)	0.015	0.46
rs77086742	55,077,348	A>G	0.132	0.127	0.61		1.07 (0.82-1.40)	0.00071	-0.87
rs6554160	55,077,549	T>A	0.217	0.178	0.0043	0.038	1.39 (1.11-1.73)	0.015	0.46
rs111944671	55,078,108	G>A	0.222	0.200	0.043	0.39	1.25 (1.01-1.56)	0.0031	-0.22
rs28436914	55,078,194	A>T	0.222	0.200	0.043	0.39	1.25 (1.01-1.56)	0.0031	-0.22
rs28753397	55,078,496	C>T	0.222	0.200	0.043	0.39	1.25 (1.01-1.56)	0.0031	-0.22
rs139145626	55,078,614	C>T	0.217	0.178	0.0043	0.038	1.39 (1.11-1.73)	0.015	0.46
rs718454	55,079,544	G>C	0.222	0.200	0.043	0.39	1.25 (1.01-1.56)	0.0031	-0.22
rs718455	55,079,614	A>C	0.222	0.200	0.043	0.39	1.25 (1.01-1.56)	0.0031	-0.22
rs12648347	55,080,051	A>G	0.222	0.200	0.043	0.39	1.25 (1.01-1.56)	0.0031	-0.22
rs12505054	55,080,371	T>G	0.222	0.200	0.043	0.39	1.25 (1.01-1.56)	0.0031	-0.22
rs7673267	55,081,133	C>T	0.222	0.200	0.043	0.39	1.25 (1.01-1.56)	0.0031	-0.22
rs11133312	55,081,744	C>T	0.222	0.200	0.043	0.39	1.25 (1.01-1.56)	0.0031	-0.22
rs11133313	55,081,757	C>T	0.222	0.200	0.043	0.39	1.25 (1.01-1.56)	0.0031	-0.22
rs11133314	55,081,850	C>A	0.222	0.200	0.043	0.39	1.25 (1.01-1.56)	0.0031	-0.22
rs11133315	55,082,158	G>A	0.217	0.178	0.0043	0.038	1.39 (1.11-1.73)	0.015	0.46
rs4398585	55,082,353	C>T	0.222	0.200	0.043	0.39	1.25 (1.01-1.56)	0.0031	-0.22
rs28441665	55,083,056	T>C	0.132	0.127	0.61		1.07 (0.82-1.40)	0.00071	-0.87
rs4864855	55,083,451	G>T	0.222	0.200	0.043	0.39	1.25 (1.01-1.56)	0.0031	-0.22
rs4864856	55,084,720	G>A	0.222	0.200	0.043	0.39	1.25 (1.01-1.56)	0.0031	-0.22
rs7683707	55,086,036	T>C	0.222	0.200	0.043	0.39	1.25 (1.01-1.56)	0.0031	-0.22
rs60806423	55,086,816	C>T	0.194	0.156	0.0062	0.055	1.39 (1.10-1.75)	0.012	0.35
rs192263827	55,087,113	T>C	0.108	0.111	0.13		0.81 (0.61-1.07)	0.0015	-0.54
rs113828936	55,087,144	G>A	0.194	0.156	0.0062	0.055	1.39 (1.10-1.75)	0.012	0.35
rs17084051	55,087,581	C>A	0.194	0.156	0.0062	0.055	1.39 (1.10-1.75)	0.012	0.35
rs28622224	55,088,093	C>T	0.326	0.283	0.0084	0.075	1.30 (1.07-1.58)	0.0093	0.25
rs61320297	55,088,175	G>A	0.194	0.156	0.0062	0.055	1.39 (1.10-1.75)	0.012	0.35
rs7673597	55,088,543	C>T	0.194	0.156	0.0062	0.055	1.39 (1.10-1.75)	0.012	0.35
rs7673625	55,088,586	C>T	0.194	0.156	0.0062	0.055	1.39 (1.10-1.75)	0.012	0.35
rs7673984	55,088,761	C>T	0.194	0.156	0.0062	0.055	1.39 (1.10-1.75)	0.012	0.35
rs4394060	55,088,909	C>A	0.194	0.156	0.0062	0.055	1.39 (1.10-1.75)	0.012	0.35
rs4864857	55,089,814	T>C	0.194	0.156	0.0062	0.055	1.39 (1.10-1.75)	0.012	0.35
rs4864858	55,089,953	T>A	0.194	0.156	0.0062	0.055	1.39 (1.10-1.75)	0.012	0.35
rs4864859	55,090,015	A>G	0.194	0.156	0.0062	0.055	1.39 (1.10-1.75)	0.012	0.35
rs4864860	55,090,021	T>C	0.194	0.156	0.0062	0.055	1.39 (1.10-1.75)	0.012	0.35
rs60863210	55,090,099	T>C	0.194	0.156	0.0062	0.055	1.39 (1.10-1.75)	0.012	0.35
rs7698425	55,090,656	C>T	0.194	0.156	0.0062	0.055	1.39 (1.10-1.75)	0.012	0.35
rs7681399	55,090,886	T>G	0.194	0.156	0.0062	0.055	1.39 (1.10-1.75)	0.012	0.35
rs2114039	55,092,626	T>C	0.326	0.284	0.011	0.095	1.29 (1.06-1.56)	0.0079	0.18
rs1800809	55,093,914	A>G	0.194	0.157	0.0081	0.073	1.37 (1.09-1.73)	0.0096	0.27
rs6554162	55,093,955	G>A	0.326	0.284	0.011	0.095	1.29 (1.06-1.56)	0.0079	0.18
rs1800810	55,094,031	C>G	0.194	0.157	0.0081	0.073	1.37 (1.09-1.73)	0.0096	0.27
rs1800813	55,094,467	G>A	0.194	0.157	0.0081	0.073	1.37 (1.09-1.73)	0.0096	0.27
rs1800812	55,094,629	G>T	0.194	0.157	0.0081	0.073	1.37 (1.09-1.73)	0.0096	0.27
rs7690503	55,096,150	C>G	0.194	0.157	0.0081	0.073	1.37 (1.09-1.73)	0.0096	0.27
rs7673027	55,096,180	T>A	0.194	0.157	0.0081	0.073	1.37 (1.09-1.73)	0.0096	0.27
rs7668190	55,096,270	A>T	0.326	0.284	0.011	0.095	1.29 (1.06-1.56)	0.0079	0.18
rs7689569	55,096,398	G>A	0.194	0.157	0.0081	0.073	1.37 (1.09-1.73)	0.0096	0.27
rs7673853	55,096,606	T>C	0.194	0.157	0.0081	0.073	1.37 (1.09-1.73)	0.0096	0.27
rs7679903	55,097,373	T>C	0.194	0.157	0.0081	0.073	1.37 (1.09-1.73)	0.0096	0.27
rs890203	55,097,405	A>C	0.194	0.157	0.0081	0.073	1.37 (1.09-1.73)	0.0096	0.27
rs4864861	55,097,685	C>T	0.194	0.157	0.0081	0.073	1.37 (1.09-1.73)	0.0096	0.27
rs4864504	55,097,835	G>C	0.326	0.284	0.011	0.095	1.29 (1.06-1.56)	0.0079	0.18
rs4608869	55,098,936	G>C	0.194	0.157	0.0081	0.073	1.37 (1.09-1.73)	0.0096	0.27
rs4368668	55,098,954	A>G	0.194	0.157	0.0081	0.073	1.37 (1.09-1.73)	0.0096	0.27
rs4635872	55,099,041	C>A	0.194	0.157	0.0081	0.073	1.37 (1.09-1.73)	0.0096	0.27
rs6850748	55,099,164	T>G	0.194	0.157	0.0081	0.073	1.37 (1.09-1.73)	0.0096	0.27
rs4864862	55,100,489	G>A	0.194	0.157	0.0081	0.073	1.37 (1.09-1.73)	0.0096	0.27
rs4864863	55,100,831	A>G	0.194	0.157	0.0081	0.073	1.37 (1.09-1.73)	0.0096	0.27
rs7678144	55,102,425	T>C	0.194	0.157	0.0081	0.073	1.37 (1.09-1.73)	0.0096	0.27

Supplementary Table S2. (Cont.)

SNP	Position on Chr. 4 (GRCh37)	Alleles (1>2)	Minor Allele Freq.		*P*	*P*c	OR (95% CI)	Fine Mapping	
			Cases (N=514)	Controls (N=842)				PIP	$Log_{10}BF$
rs6554163	55,102,559	T>A	0.194	0.157	0.0081	0.073	1.37 (1.09-1.73)	0.0096	0.27
rs6836215	55,102,741	T>C	0.194	0.157	0.0081	0.073	1.37 (1.09-1.73)	0.0096	0.27
rs67432867	55,104,454	A>T	0.194	0.157	0.0081	0.073	1.37 (1.09-1.73)	0.0096	0.27
rs73252942	55,104,604	T>C	0.194	0.157	0.0081	0.073	1.37 (1.09-1.73)	0.0096	0.27
rs6554164	55,106,695	T>C	0.326	0.283	0.010	0.091	1.29 (1.06-1.57)	0.0081	0.19
rs35714192	55,107,750	G>C	0.460	0.437	0.47		1.07 (0.89-1.28)	0.00078	-0.83
rs147558377	55,113,623	C>G	0.192	0.156	0.0082	0.073	1.37 (1.09-1.73)	0.0096	0.27
rs13435164	55,115,828	C>G	0.325	0.283	0.011	0.099	1.29 (1.06-1.56)	0.0077	0.17
rs6554165	55,115,847	G>A	0.325	0.283	0.011	0.099	1.29 (1.06-1.56)	0.0077	0.17
rs79938737	55,116,048	G>C	0.131	0.126	0.63		1.07 (0.82-1.40)	0.00070	-0.87
rs60251664	55,117,805	T>C	0.131	0.126	0.63		1.07 (0.82-1.40)	0.00070	-0.87
rs76091088	55,119,631	T>C	0.108	0.111	0.13		0.81 (0.61-1.07)	0.0015	-0.54
rs4422471	55,121,921	C>T	0.190	0.154	0.0085	0.076	1.37 (1.08-1.73)	0.0093	0.26
rs10029499	55,122,494	A>G	0.323	0.281	0.010	0.093	1.29 (1.06-1.57)	0.0080	0.19
rs7677751	55,124,460	C>T	0.186	0.150	0.0098	0.088	1.37 (1.08-1.73)	0.0085	0.21
rs9991165	55,124,591	A>G	0.180	0.146	0.011	0.10	1.36 (1.07-1.72)	0.0076	0.17
rs2303429	55,124,870	C>T	0.108	0.111	0.13		0.81 (0.61-1.07)	0.0015	-0.54
rs2303430	55,125,058	A>T	0.459	0.471	0.48		1.06 (0.90-1.27)	0.00078	-0.83
rs73252946	55,125,992	G>A	0.178	0.146	0.016	0.14	1.34 (1.06-1.70)	0.0061	0.07
rs58435984	55,127,990	T>C	0.178	0.146	0.016	0.14	1.34 (1.06-1.70)	0.0061	0.07
rs2229307	55,130,078	T>C	0.178	0.146	0.016	0.14	1.34 (1.06-1.70)	0.0061	0.07
rs2307049	55,130,154	G>A	0.178	0.146	0.016	0.14	1.34 (1.06-1.70)	0.0061	0.07
rs67279506	55,130,682	A>G	0.178	0.146	0.016	0.14	1.34 (1.06-1.70)	0.0061	0.07
rs58727676	55,130,794	T>G	0.178	0.146	0.016	0.14	1.34 (1.06-1.70)	0.0061	0.07
rs7688997	55,131,450	C>A	0.178	0.146	0.016	0.14	1.34 (1.06-1.70)	0.0061	0.07
rs56145315	55,131,689	C>T	0.178	0.146	0.016	0.14	1.34 (1.06-1.70)	0.0061	0.07
rs28600756	55,131,732	C>A	0.178	0.146	0.016	0.14	1.34 (1.06-1.70)	0.0061	0.07
rs67600360	55,132,162	A>G	0.178	0.146	0.016	0.14	1.34 (1.06-1.70)	0.0061	0.07
rs4864864	55,132,325	C>T	0.178	0.146	0.016	0.14	1.34 (1.06-1.70)	0.0061	0.07
rs4864865	55,132,878	T>A	0.178	0.146	0.016	0.14	1.34 (1.06-1.70)	0.0061	0.07
rs73252948	55,133,252	T>C	0.178	0.146	0.016	0.14	1.34 (1.06-1.70)	0.0061	0.07
rs4358459	55,133,726	T>G	0.178	0.146	0.016	0.14	1.34 (1.06-1.70)	0.0061	0.07
rs28489067	55,133,936	C>T	0.178	0.146	0.016	0.14	1.34 (1.06-1.70)	0.0061	0.07
rs28650939	55,133,959	C>T	0.178	0.146	0.016	0.14	1.34 (1.06-1.70)	0.0061	0.07
rs28528897	55,134,339	C>G	0.178	0.146	0.016	0.14	1.34 (1.06-1.70)	0.0061	0.07
rs7660560	55,134,394	G>A	0.178	0.146	0.016	0.14	1.34 (1.06-1.70)	0.0061	0.07
rs7691129	55,134,466	T>C	0.178	0.146	0.016	0.14	1.34 (1.06-1.70)	0.0061	0.07
rs7686588	55,134,628	A>G	0.178	0.146	0.016	0.14	1.34 (1.06-1.70)	0.0061	0.07
rs12641563	55,135,447	C>G	0.178	0.146	0.016	0.14	1.34 (1.06-1.70)	0.0061	0.07
rs12644709	55,135,580	A>G	0.178	0.146	0.016	0.14	1.34 (1.06-1.70)	0.0061	0.07
rs12644749	55,135,771	A>G	0.178	0.146	0.016	0.14	1.34 (1.06-1.70)	0.0061	0.07
rs12508225	55,136,027	G>C	0.178	0.146	0.016	0.14	1.34 (1.06-1.70)	0.0061	0.07
rs12506290	55,136,132	T>A	0.178	0.146	0.016	0.14	1.34 (1.06-1.70)	0.0061	0.07
rs12505491	55,136,245	A>G	0.178	0.146	0.016	0.14	1.34 (1.06-1.70)	0.0061	0.07
rs1565669	55,137,183	T>C	0.178	0.146	0.016	0.14	1.34 (1.06-1.70)	0.0061	0.07
rs1565670	55,137,281	A>G	0.178	0.146	0.016	0.14	1.34 (1.06-1.70)	0.0061	0.07
rs35597368	55,139,771	T>C	0.169	0.139	0.021	0.19	1.33 (1.04-1.69)	0.0050	-0.02
rs869978	55,140,016	C>T	0.288	0.256	0.038	0.34	1.24 (1.01-1.51)	0.0034	-0.19
rs2307050	55,141,293	G>A	0.169	0.139	0.021	0.19	1.33 (1.04-1.69)	0.0050	-0.02
rs73252950	55,141,500	C>T	0.170	0.139	0.020	0.18	1.33 (1.05-1.70)	0.0053	0.00
rs6855761	55,141,532	A>T	0.170	0.139	0.020	0.18	1.33 (1.05-1.70)	0.0053	0.00
rs7656613	55,141,843	T>C	0.455	0.464	0.36		1.08 (0.91-1.29)	0.00088	-0.77
rs1105825	55,142,205	G>A	0.169	0.138	0.019	0.17	1.34 (1.05-1.70)	0.0054	0.01
rs67388297	55,142,691	C>G	0.170	0.138	0.018	0.16	1.34 (1.05-1.71)	0.0056	0.03
rs28698464	55,143,110	A>G	0.170	0.137	0.017	0.15	1.34 (1.05-1.71)	0.0058	0.05
rs1316926	55,143,286	G>A	0.236	0.258	0.082		0.84 (0.68-1.02)	0.0020	-0.41
rs28374326	55,143,321	C>T	0.170	0.138	0.023	0.21	1.32 (1.04-1.69)	0.0047	-0.05
rs10028020	55,143,577	G>A	0.171	0.138	0.023	0.21	1.33 (1.04-1.69)	0.0047	-0.04
rs1907819	55,144,420	G>A	0.179	0.145	0.032	0.29	1.29 (1.02-1.64)	0.0038	-0.14
rs2412556	55,145,258	A>G	0.178	0.145	0.038	0.34	1.29 (1.01-1.63)	0.0034	-0.19
rs4289498	55,145,432	A>G	0.178	0.145	0.038	0.34	1.29 (1.01-1.63)	0.0034	-0.19
rs1547904	55,146,389	C>T	0.178	0.145	0.038	0.34	1.29 (1.01-1.63)	0.0034	-0.19
rs1547905	55,146,754	C>A	0.178	0.145	0.038	0.34	1.29 (1.01-1.63)	0.0034	-0.19
rs3816888	55,146,761	C>T	0.020	0.021	0.55		1.21 (0.65-2.27)	0.00074	-0.85
rs58025349	55,146,927	A>T	0.178	0.145	0.038	0.34	1.29 (1.01-1.63)	0.0034	-0.19
rs2291591	55,147,769	C>T	0.089	0.093	0.87		0.97 (0.71-1.33)	0.00065	-0.91
rs28811736	55,148,547	C>G	0.178	0.145	0.038	0.34	1.29 (1.01-1.63)	0.0034	-0.19

Supplementary Table S2. (Cont.)

SNP	Position on Chr. 4 (GRCh37)	Alleles (1>2)	Minor Allele Freq.		*P*	*P* c	OR (95% CI)	Fine Mapping	
			Cases (N=514)	Controls (N=842)				PIP	$Log_{10}BF$
rs7677708	55,149,258	A>G	0.178	0.145	0.038	0.34	1.29 (1.01-1.63)	0.0034	-0.19
rs2412557	55,149,457	A>C	0.178	0.145	0.038	0.34	1.29 (1.01-1.63)	0.0034	-0.19
rs2412558	55,149,507	A>G	0.178	0.145	0.038	0.34	1.29 (1.01-1.63)	0.0034	-0.19
rs2228230	55,152,040	C>T	0.174	0.144	0.055		1.26 (1.00-1.61)	0.0027	-0.29
rs4864872	55,152,284	G>T	0.174	0.144	0.055		1.26 (1.00-1.61)	0.0027	-0.29
rs11940889	55,152,583	C>T	0.174	0.144	0.055		1.26 (1.00-1.61)	0.0027	-0.29
rs11931555	55,152,715	G>C	0.175	0.144	0.043	0.39	1.28 (1.01-1.63)	0.0031	-0.22
rs10020847	55,153,134	C>T	0.174	0.144	0.055		1.26 (1.00-1.61)	0.0027	-0.29
rs144143098	55,153,551	A>G	0.023	0.021	0.46		1.25 (0.69-2.25)	0.00079	-0.82
rs10021728	55,154,109	C>T	0.174	0.144	0.055		1.26 (1.00-1.61)	0.0027	-0.29
rs9993187	55,154,250	T>C	0.174	0.144	0.055		1.26 (1.00-1.61)	0.0027	-0.29
rs11733839	55,154,527	G>C	0.174	0.144	0.055		1.26 (1.00-1.61)	0.0027	-0.29
rs55732997	55,154,891	T>C	0.174	0.144	0.055		1.26 (1.00-1.61)	0.0027	-0.29
rs10004857	55,155,980	G>A	0.174	0.144	0.055		1.26 (1.00-1.61)	0.0027	-0.29
rs11133317	55,156,091	G>T	0.174	0.144	0.055		1.26 (1.00-1.61)	0.0027	-0.29
rs4864875	55,156,166	A>G	0.174	0.141	0.029	0.26	1.31 (1.03-1.66)	0.0040	-0.11
rs4864876	55,156,300	C>T	0.174	0.141	0.029	0.26	1.31 (1.03-1.66)	0.0040	-0.11
rs2276948	55,156,400	G>A	0.174	0.141	0.029	0.26	1.31 (1.03-1.66)	0.0040	-0.11
rs13147194	55,157,134	T>G	0.174	0.141	0.029	0.26	1.31 (1.03-1.66)	0.0040	-0.11
rs10010509	55,157,206	G>T	0.174	0.141	0.029	0.26	1.31 (1.03-1.66)	0.0040	-0.11
rs10003055	55,157,666	T>G	0.174	0.141	0.029	0.26	1.31 (1.03-1.66)	0.0040	-0.11
rs11935157	55,158,514	A>T	0.172	0.140	0.034	0.31	1.30 (1.02-1.65)	0.0036	-0.16
rs139471049	55,158,597	A>G	0.086	0.090	0.91		0.98 (0.71-1.35)	0.00064	-0.91
rs60218083	55,158,938	A>G	0.169	0.139	0.046	0.41	1.28 (1.01-1.63)	0.0030	-0.24
rs55784333	55,159,391	C>G	0.169	0.139	0.046	0.41	1.28 (1.01-1.63)	0.0030	-0.24
rs10032688	55,160,658	G>A	0.169	0.139	0.046	0.41	1.28 (1.01-1.63)	0.0030	-0.24
rs3733540	55,161,254	T>C	0.169	0.139	0.046	0.41	1.28 (1.01-1.63)	0.0030	-0.24
rs3690	55,161,813	A>C	0.169	0.139	0.046	0.41	1.28 (1.01-1.63)	0.0030	-0.24
rs17739921	55,164,866	A>C	0.264	0.285	0.079		0.84 (0.69-1.02)	0.0021	-0.40
rs34491905	55,165,075	C>T	0.263	0.283	0.089		0.85 (0.70-1.03)	0.0019	-0.43
rs1961871	55,167,266	G>A	0.349	0.357	0.57		1.05 (0.88-1.27)	0.00073	-0.86
rs1826426	55,167,287	A>G	0.400	0.403	0.27		1.11 (0.92-1.32)	0.0010	-0.71
rs10000267	55,167,931	A>G	0.370	0.374	0.39		1.08 (0.90-1.30)	0.00085	-0.79
rs11722786	55,169,182	C>T	0.349	0.357	0.57		1.05 (0.88-1.27)	0.00073	-0.86
rs11727002	55,169,183	A>G	0.349	0.357	0.57		1.05 (0.88-1.27)	0.00073	-0.86
rs55836714	55,170,352	T>C	0.370	0.374	0.39		1.08 (0.90-1.30)	0.00085	-0.79
rs77890222	55,170,462	C>A	0.349	0.357	0.57		1.05 (0.88-1.27)	0.00073	-0.86
rs62299407	55,171,129	A>G	0.349	0.357	0.57		1.05 (0.88-1.27)	0.00073	-0.86
rs4594778	55,172,289	C>T	0.349	0.357	0.57		1.05 (0.88-1.27)	0.00073	-0.86
rs4864877	55,172,357	A>G	0.349	0.357	0.57		1.05 (0.88-1.27)	0.00073	-0.86
rs13353653	55,172,713	G>A	0.349	0.357	0.57		1.05 (0.88-1.27)	0.00073	-0.86
rs4864878	55,173,715	C>T	0.349	0.357	0.57		1.05 (0.88-1.27)	0.00073	-0.86
rs6858442	55,173,933	G>A	0.349	0.357	0.57		1.05 (0.88-1.27)	0.00073	-0.86
rs6554168	55,174,827	G>A	0.349	0.357	0.57		1.05 (0.88-1.27)	0.00073	-0.86
rs6554170	55,174,885	C>T	0.349	0.357	0.57		1.05 (0.88-1.27)	0.00073	-0.86
rs1565668	55,175,601	T>C	0.349	0.357	0.57		1.05 (0.88-1.27)	0.00073	-0.86
rs1565665	55,175,768	C>G	0.349	0.357	0.57		1.05 (0.88-1.27)	0.00073	-0.86
rs28889275	55,176,073	G>A	0.349	0.357	0.57		1.05 (0.88-1.27)	0.00073	-0.86
rs28884174	55,176,383	T>C	0.349	0.357	0.57		1.05 (0.88-1.27)	0.00073	-0.86
rs28707743	55,178,018	C>T	0.347	0.356	0.58		1.05 (0.88-1.26)	0.00072	-0.86
rs28547147	55,179,059	C>A	0.349	0.357	0.56		1.06 (0.88-1.27)	0.00073	-0.85
rs10016845	55,183,077	T>C	0.347	0.356	0.58		1.05 (0.88-1.26)	0.00072	-0.86
rs11732117	55,184,119	A>G	0.347	0.356	0.58		1.05 (0.88-1.26)	0.00072	-0.86
rs6850695	55,185,035	C>T	0.347	0.356	0.58		1.05 (0.88-1.26)	0.00072	-0.86
rs10028483	55,186,168	T>C	0.347	0.356	0.58		1.05 (0.88-1.26)	0.00072	-0.86
rs9991681	55,186,390	G>A	0.346	0.356	0.61		1.05 (0.87-1.26)	0.00071	-0.87
rs28714526	55,186,508	A>G	0.347	0.356	0.58		1.05 (0.88-1.26)	0.00072	-0.86
rs2087806	55,186,956	C>T	0.347	0.356	0.58		1.05 (0.88-1.26)	0.00072	-0.86
rs10029431	55,187,406	A>T	0.346	0.356	0.61		1.05 (0.87-1.26)	0.00071	-0.87

SNP, single-nucleotide polymorphism; 1, major allele; 2, minor allele; *P* c, corrected *P* -value; OR, odds ratio; CI, confidence interval; PIP, posterior inclusion probability; $Log_{10}BF$, log_{10} Bayes factor.

Supplementary Table S3. Association and fine mapping results for 192 SNPs in the *PDGFRA* gene region in the cases with corneal astigmatism ≤ − 0.75 D

			Minor Allele Freq.				Fine Mapping	
SNP	Position on Chr. 4 (GRCh37)	Alleles (1>2)	Cases (N=1535)	Controls (N=842)	*P*	OR (95% CI)	PIP	$Log_{10}BF$
rs17084040	55,074,368	G>A	0.123	0.127	0.92	0.99 (0.82-1.20)	0.0022	-0.38
rs17084042	55,074,542	G>T	0.123	0.127	0.92	0.99 (0.82-1.20)	0.0022	-0.38
rs57217483	55,074,922	G>T	0.123	0.127	0.92	0.99 (0.82-1.20)	0.0022	-0.38
rs28431840	55,075,792	T>G	0.123	0.127	0.92	0.99 (0.82-1.20)	0.0022	-0.38
rs6832597	55,076,237	C>A	0.196	0.178	0.16	1.12 (0.96-1.32)	0.0051	-0.01
rs6831380	55,076,345	G>A	0.196	0.178	0.16	1.12 (0.96-1.32)	0.0051	-0.01
rs7676972	55,077,078	C>T	0.196	0.178	0.16	1.12 (0.96-1.32)	0.0051	-0.01
rs77086742	55,077,348	A>G	0.123	0.127	0.92	0.99 (0.82-1.20)	0.0022	-0.38
rs6554160	55,077,549	T>A	0.196	0.178	0.16	1.12 (0.96-1.32)	0.0051	-0.01
rs111944671	55,078,108	G>A	0.207	0.200	0.48	1.06 (0.90-1.24)	0.0027	-0.29
rs28436914	55,078,194	A>T	0.207	0.200	0.48	1.06 (0.90-1.24)	0.0027	-0.29
rs28753397	55,078,496	C>T	0.207	0.200	0.48	1.06 (0.90-1.24)	0.0027	-0.29
rs139145626	55,078,614	C>T	0.196	0.178	0.16	1.12 (0.96-1.32)	0.0051	-0.01
rs718454	55,079,544	G>C	0.207	0.200	0.48	1.06 (0.90-1.24)	0.0027	-0.29
rs718455	55,079,614	A>C	0.207	0.200	0.48	1.06 (0.90-1.24)	0.0027	-0.29
rs12648347	55,080,051	A>G	0.207	0.200	0.48	1.06 (0.90-1.24)	0.0027	-0.29
rs12505054	55,080,371	T>G	0.207	0.200	0.48	1.06 (0.90-1.24)	0.0027	-0.29
rs7673267	55,081,133	C>T	0.207	0.200	0.48	1.06 (0.90-1.24)	0.0027	-0.29
rs11133312	55,081,744	C>T	0.207	0.200	0.48	1.06 (0.90-1.24)	0.0027	-0.29
rs11133313	55,081,757	C>T	0.207	0.200	0.48	1.06 (0.90-1.24)	0.0027	-0.29
rs11133314	55,081,850	C>A	0.207	0.200	0.48	1.06 (0.90-1.24)	0.0027	-0.29
rs11133315	55,082,158	G>A	0.196	0.178	0.16	1.12 (0.96-1.32)	0.0051	-0.01
rs4398585	55,082,353	C>T	0.207	0.200	0.48	1.06 (0.90-1.24)	0.0027	-0.29
rs28441665	55,083,056	T>C	0.123	0.127	0.92	0.99 (0.82-1.20)	0.0022	-0.38
rs4864855	55,083,451	G>T	0.207	0.200	0.48	1.06 (0.90-1.24)	0.0027	-0.29
rs4864856	55,084,720	G>A	0.207	0.200	0.48	1.06 (0.90-1.24)	0.0027	-0.29
rs7683707	55,086,036	T>C	0.207	0.200	0.48	1.06 (0.90-1.24)	0.0027	-0.29
rs60806423	55,086,816	C>T	0.177	0.156	0.079	1.17 (0.98-1.38)	0.0081	0.19
rs192263827	55,087,113	T>C	0.107	0.111	0.44	0.92 (0.76-1.13)	0.0028	-0.27
rs113828936	55,087,144	G>A	0.177	0.156	0.079	1.17 (0.98-1.38)	0.0081	0.19
rs17084051	55,087,581	C>A	0.177	0.156	0.079	1.17 (0.98-1.38)	0.0081	0.19
rs28622224	55,088,093	C>T	0.300	0.283	0.17	1.10 (0.96-1.27)	0.0049	-0.03
rs61320297	55,088,175	G>A	0.177	0.156	0.079	1.17 (0.98-1.38)	0.0081	0.19
rs7673597	55,088,543	C>T	0.177	0.156	0.079	1.17 (0.98-1.38)	0.0081	0.19
rs7673625	55,088,586	C>T	0.177	0.156	0.079	1.17 (0.98-1.38)	0.0081	0.19
rs7673984	55,088,761	C>T	0.177	0.156	0.079	1.17 (0.98-1.38)	0.0081	0.19
rs4394060	55,088,909	C>A	0.177	0.156	0.079	1.17 (0.98-1.38)	0.0081	0.19
rs4864857	55,089,814	T>C	0.177	0.156	0.079	1.17 (0.98-1.38)	0.0081	0.19
rs4864858	55,089,953	T>A	0.177	0.156	0.079	1.17 (0.98-1.38)	0.0081	0.19
rs4864859	55,090,015	A>G	0.177	0.156	0.079	1.17 (0.98-1.38)	0.0081	0.19
rs4864860	55,090,021	T>C	0.177	0.156	0.079	1.17 (0.98-1.38)	0.0081	0.19
rs60863210	55,090,099	T>C	0.177	0.156	0.079	1.17 (0.98-1.38)	0.0081	0.19
rs7698425	55,090,656	C>T	0.177	0.156	0.079	1.17 (0.98-1.38)	0.0081	0.19
rs7681399	55,090,886	T>G	0.177	0.156	0.079	1.17 (0.98-1.38)	0.0081	0.19
rs2114039	55,092,626	T>C	0.301	0.284	0.19	1.10 (0.96-1.26)	0.0046	-0.06
rs1800809	55,093,914	A>G	0.177	0.157	0.094	1.16 (0.97-1.37)	0.0072	0.14
rs6554162	55,093,955	G>A	0.301	0.284	0.19	1.10 (0.96-1.26)	0.0046	-0.06
rs1800810	55,094,031	C>G	0.177	0.157	0.094	1.16 (0.97-1.37)	0.0072	0.14
rs1800813	55,094,467	G>A	0.177	0.157	0.094	1.16 (0.97-1.37)	0.0072	0.14
rs1800812	55,094,629	G>T	0.177	0.157	0.094	1.16 (0.97-1.37)	0.0072	0.14
rs7690503	55,096,150	C>G	0.177	0.157	0.094	1.16 (0.97-1.37)	0.0072	0.14
rs7673027	55,096,180	T>A	0.177	0.157	0.094	1.16 (0.97-1.37)	0.0072	0.14
rs7668190	55,096,270	A>T	0.301	0.284	0.19	1.10 (0.96-1.26)	0.0046	-0.06
rs7689569	55,096,398	G>A	0.177	0.157	0.094	1.16 (0.97-1.37)	0.0072	0.14
rs7673853	55,096,606	T>C	0.177	0.157	0.094	1.16 (0.97-1.37)	0.0072	0.14
rs7679903	55,097,373	T>C	0.177	0.157	0.094	1.16 (0.97-1.37)	0.0072	0.14
rs890203	55,097,405	A>C	0.177	0.157	0.094	1.16 (0.97-1.37)	0.0072	0.14
rs4864861	55,097,685	C>T	0.177	0.157	0.094	1.16 (0.97-1.37)	0.0072	0.14
rs4864504	55,097,835	G>C	0.301	0.284	0.19	1.10 (0.96-1.26)	0.0046	-0.06
rs4608869	55,098,936	G>C	0.177	0.157	0.094	1.16 (0.97-1.37)	0.0072	0.14
rs4368668	55,098,954	A>G	0.177	0.157	0.094	1.16 (0.97-1.37)	0.0072	0.14
rs4635872	55,099,041	C>A	0.177	0.157	0.094	1.16 (0.97-1.37)	0.0072	0.14
rs6850748	55,099,164	T>G	0.177	0.157	0.094	1.16 (0.97-1.37)	0.0072	0.14
rs4864862	55,100,489	G>A	0.177	0.157	0.094	1.16 (0.97-1.37)	0.0072	0.14
rs4864863	55,100,831	A>G	0.177	0.157	0.094	1.16 (0.97-1.37)	0.0072	0.14
rs7678144	55,102,425	T>C	0.177	0.157	0.094	1.16 (0.97-1.37)	0.0072	0.14

Supplementary Table S3. (Cont.)

SNP	Position on Chr. 4 (GRCh37)	Alleles (1>2)	Minor Allele Freq.		*P*	OR (95% CI)	Fine Mapping	
			Cases (N=1535)	Controls (N=842)			PIP	$Log_{10}BF$
rs6554163	55,102,559	T>A	0.177	0.157	0.094	1.16 (0.97-1.37)	0.0072	0.14
rs6836215	55,102,741	T>C	0.177	0.157	0.094	1.16 (0.97-1.37)	0.0072	0.14
rs67432867	55,104,454	A>T	0.177	0.157	0.094	1.16 (0.97-1.37)	0.0072	0.14
rs73252942	55,104,604	T>C	0.178	0.157	0.081	1.16 (0.98-1.38)	0.0079	0.18
rs6554164	55,106,695	T>C	0.301	0.283	0.17	1.10 (0.96-1.27)	0.0049	-0.03
rs35714192	55,107,750	G>C	0.451	0.437	0.40	1.06 (0.93-1.20)	0.0030	-0.25
rs147558377	55,113,623	C>G	0.177	0.156	0.077	1.17 (0.98-1.39)	0.0082	0.20
rs13435164	55,115,828	C>G	0.300	0.283	0.18	1.10 (0.96-1.26)	0.0047	-0.04
rs6554165	55,115,847	G>A	0.300	0.283	0.18	1.10 (0.96-1.26)	0.0047	-0.04
rs79938737	55,116,048	G>C	0.123	0.126	0.93	0.99 (0.82-1.20)	0.0022	-0.38
rs60251664	55,117,805	T>C	0.123	0.126	0.93	0.99 (0.82-1.20)	0.0022	-0.38
rs76091088	55,119,631	T>C	0.107	0.111	0.44	0.92 (0.76-1.13)	0.0028	-0.27
rs4422471	55,121,921	C>T	0.176	0.154	0.067	1.17 (0.99-1.39)	0.0090	0.24
rs10029499	55,122,494	A>G	0.299	0.281	0.15	1.11 (0.96-1.27)	0.0052	0.00
rs7677751	55,124,460	C>T	0.173	0.150	0.063	1.18 (0.99-1.40)	0.0095	0.26
rs9991165	55,124,591	A>G	0.167	0.146	0.073	1.17 (0.98-1.40)	0.0085	0.21
rs2303429	55,124,870	C>T	0.107	0.111	0.44	0.92 (0.76-1.13)	0.0028	-0.27
rs2303430	55,125,058	A>T	0.466	0.471	0.99	1.00 (0.88-1.13)	0.0022	-0.38
rs73252946	55,125,992	G>A	0.166	0.146	0.091	1.16 (0.98-1.38)	0.0073	0.15
rs58435984	55,127,990	T>C	0.166	0.146	0.091	1.16 (0.98-1.38)	0.0073	0.15
rs2229307	55,130,078	T>C	0.166	0.146	0.091	1.16 (0.98-1.38)	0.0073	0.15
rs2307049	55,130,154	G>A	0.166	0.146	0.091	1.16 (0.98-1.38)	0.0073	0.15
rs67279506	55,130,682	A>G	0.166	0.146	0.091	1.16 (0.98-1.38)	0.0073	0.15
rs58727676	55,130,794	T>G	0.166	0.146	0.091	1.16 (0.98-1.38)	0.0073	0.15
rs7688997	55,131,450	C>A	0.166	0.146	0.091	1.16 (0.98-1.38)	0.0073	0.15
rs56145315	55,131,689	C>T	0.166	0.146	0.091	1.16 (0.98-1.38)	0.0073	0.15
rs28600756	55,131,732	C>A	0.166	0.146	0.091	1.16 (0.98-1.38)	0.0073	0.15
rs67600360	55,132,162	A>G	0.166	0.146	0.091	1.16 (0.98-1.38)	0.0073	0.15
rs4864864	55,132,325	C>T	0.166	0.146	0.091	1.16 (0.98-1.38)	0.0073	0.15
rs4864865	55,132,878	T>A	0.166	0.146	0.091	1.16 (0.98-1.38)	0.0073	0.15
rs73252948	55,133,252	T>C	0.166	0.146	0.091	1.16 (0.98-1.38)	0.0073	0.15
rs4358459	55,133,726	T>G	0.166	0.146	0.091	1.16 (0.98-1.38)	0.0073	0.15
rs28489067	55,133,936	C>T	0.166	0.146	0.091	1.16 (0.98-1.38)	0.0073	0.15
rs28650939	55,133,959	C>T	0.166	0.146	0.091	1.16 (0.98-1.38)	0.0073	0.15
rs28528897	55,134,339	C>G	0.166	0.146	0.091	1.16 (0.98-1.38)	0.0073	0.15
rs7660560	55,134,394	G>A	0.166	0.146	0.091	1.16 (0.98-1.38)	0.0073	0.15
rs7691129	55,134,466	T>C	0.166	0.146	0.091	1.16 (0.98-1.38)	0.0073	0.15
rs7686588	55,134,628	A>G	0.166	0.146	0.091	1.16 (0.98-1.38)	0.0073	0.15
rs12641563	55,135,447	C>G	0.166	0.146	0.091	1.16 (0.98-1.38)	0.0073	0.15
rs12644709	55,135,580	A>G	0.166	0.146	0.091	1.16 (0.98-1.38)	0.0073	0.15
rs12644749	55,135,771	A>G	0.166	0.146	0.091	1.16 (0.98-1.38)	0.0073	0.15
rs12508225	55,136,027	G>C	0.166	0.146	0.091	1.16 (0.98-1.38)	0.0073	0.15
rs12506290	55,136,132	T>A	0.166	0.146	0.091	1.16 (0.98-1.38)	0.0073	0.15
rs12505491	55,136,245	A>G	0.166	0.146	0.091	1.16 (0.98-1.38)	0.0073	0.15
rs1565669	55,137,183	T>C	0.166	0.146	0.091	1.16 (0.98-1.38)	0.0073	0.15
rs1565670	55,137,281	A>G	0.166	0.146	0.091	1.16 (0.98-1.38)	0.0073	0.15
rs35597368	55,139,771	T>C	0.155	0.139	0.13	1.15 (0.96-1.37)	0.0056	0.04
rs869978	55,140,016	C>T	0.265	0.256	0.35	1.07 (0.93-1.24)	0.0032	-0.22
rs2307050	55,141,293	G>A	0.155	0.139	0.13	1.15 (0.96-1.37)	0.0056	0.04
rs73252950	55,141,500	C>T	0.155	0.139	0.13	1.15 (0.96-1.37)	0.0057	0.04
rs6855761	55,141,532	A>T	0.155	0.139	0.13	1.15 (0.96-1.37)	0.0057	0.04
rs7656613	55,141,843	T>C	0.460	0.464	0.93	1.01 (0.89-1.14)	0.0022	-0.38
rs1105825	55,142,205	G>A	0.152	0.138	0.17	1.13 (0.95-1.35)	0.0049	-0.03
rs67388297	55,142,691	C>G	0.153	0.138	0.17	1.13 (0.95-1.36)	0.0049	-0.02
rs28698464	55,143,110	A>G	0.153	0.137	0.14	1.14 (0.95-1.36)	0.0054	0.01
rs1316926	55,143,286	G>A	0.242	0.258	0.22	0.91 (0.79-1.06)	0.0041	-0.10
rs28374326	55,143,321	C>T	0.154	0.138	0.13	1.15 (0.96-1.37)	0.0057	0.04
rs10028020	55,143,577	G>A	0.155	0.138	0.14	1.14 (0.96-1.37)	0.0055	0.02
rs1907819	55,144,420	G>A	0.161	0.145	0.20	1.12 (0.94-1.34)	0.0044	-0.07
rs2412556	55,145,258	A>G	0.160	0.145	0.21	1.12 (0.94-1.33)	0.0043	-0.08
rs4289498	55,145,432	A>G	0.160	0.145	0.21	1.12 (0.94-1.33)	0.0043	-0.08
rs1547904	55,146,389	C>T	0.160	0.145	0.21	1.12 (0.94-1.33)	0.0043	-0.08
rs1547905	55,146,754	C>A	0.160	0.145	0.21	1.12 (0.94-1.33)	0.0043	-0.08
rs3816888	55,146,761	C>T	0.022	0.021	0.69	1.09 (0.71-1.69)	0.0023	-0.35
rs58025349	55,146,927	A>T	0.160	0.145	0.21	1.12 (0.94-1.33)	0.0043	-0.08
rs2291591	55,147,769	C>T	0.083	0.093	0.36	0.90 (0.72-1.12)	0.0031	-0.22
rs28811736	55,148,547	C>G	0.160	0.145	0.21	1.12 (0.94-1.33)	0.0043	-0.08

Supplementary Table S3. (Cont.)

SNP	Position on Chr. 4 (GRCh37)	Alleles (1>2)	Minor Allele Freq. Cases (N=1535)	Minor Allele Freq. Controls (N=842)	*P*	OR (95% CI)	Fine Mapping PIP	Fine Mapping $Log_{10}BF$
rs7677708	55,149,258	A>G	0.160	0.145	0.21	1.12 (0.94-1.33)	0.0043	-0.08
rs2412557	55,149,457	A>C	0.160	0.145	0.21	1.12 (0.94-1.33)	0.0043	-0.08
rs2412558	55,149,507	A>G	0.160	0.145	0.21	1.12 (0.94-1.33)	0.0043	-0.08
rs2228230	55,152,040	C>T	0.158	0.144	0.25	1.11 (0.93-1.32)	0.0038	-0.14
rs4864872	55,152,284	G>T	0.158	0.144	0.25	1.11 (0.93-1.32)	0.0038	-0.14
rs11940889	55,152,583	C>T	0.158	0.144	0.25	1.11 (0.93-1.32)	0.0038	-0.14
rs11931555	55,152,715	G>C	0.158	0.144	0.22	1.12 (0.94-1.33)	0.0042	-0.10
rs10020847	55,153,134	C>T	0.158	0.144	0.25	1.11 (0.93-1.32)	0.0038	-0.14
rs144143098	55,153,551	A>G	0.019	0.021	0.48	0.85 (0.55-1.33)	0.0027	-0.28
rs10021728	55,154,109	C>T	0.158	0.144	0.25	1.11 (0.93-1.32)	0.0038	-0.14
rs9993187	55,154,250	T>C	0.158	0.144	0.25	1.11 (0.93-1.32)	0.0038	-0.14
rs11733839	55,154,527	G>C	0.158	0.144	0.25	1.11 (0.93-1.32)	0.0038	-0.14
rs55732997	55,154,891	T>C	0.158	0.144	0.25	1.11 (0.93-1.32)	0.0038	-0.14
rs10004857	55,155,980	G>A	0.158	0.144	0.25	1.11 (0.93-1.32)	0.0038	-0.14
rs11133317	55,156,091	G>T	0.158	0.144	0.25	1.11 (0.93-1.32)	0.0038	-0.14
rs4864875	55,156,166	A>G	0.156	0.141	0.19	1.13 (0.94-1.35)	0.0046	-0.06
rs4864876	55,156,300	C>T	0.156	0.141	0.19	1.13 (0.94-1.35)	0.0046	-0.06
rs2276948	55,156,400	G>A	0.156	0.141	0.19	1.13 (0.94-1.35)	0.0046	-0.06
rs13147194	55,157,134	T>G	0.156	0.141	0.19	1.13 (0.94-1.35)	0.0046	-0.06
rs10010509	55,157,206	G>T	0.156	0.141	0.19	1.13 (0.94-1.35)	0.0046	-0.06
rs10003055	55,157,666	T>G	0.156	0.141	0.19	1.12 (0.94-1.34)	0.0045	-0.07
rs11935157	55,158,514	A>T	0.155	0.140	0.19	1.13 (0.94-1.35)	0.0045	-0.06
rs139471049	55,158,597	A>G	0.080	0.090	0.35	0.90 (0.72-1.12)	0.0032	-0.21
rs60218083	55,158,938	A>G	0.154	0.139	0.20	1.12 (0.94-1.34)	0.0044	-0.07
rs55784333	55,159,391	C>G	0.154	0.139	0.20	1.12 (0.94-1.34)	0.0044	-0.07
rs10032688	55,160,658	G>A	0.154	0.139	0.20	1.12 (0.94-1.34)	0.0044	-0.07
rs3733540	55,161,254	T>C	0.154	0.139	0.20	1.12 (0.94-1.34)	0.0044	-0.07
rs3690	55,161,813	A>C	0.154	0.139	0.20	1.12 (0.94-1.34)	0.0044	-0.07
rs17739921	55,164,866	A>C	0.275	0.285	0.35	0.94 (0.81-1.07)	0.0032	-0.21
rs34491905	55,165,075	C>T	0.275	0.283	0.42	0.94 (0.82-1.08)	0.0029	-0.26
rs1961871	55,167,266	G>A	0.366	0.357	0.22	1.08 (0.95-1.23)	0.0041	-0.10
rs1826426	55,167,287	A>G	0.411	0.403	0.21	1.08 (0.96-1.23)	0.0043	-0.09
rs10000267	55,167,931	A>G	0.384	0.374	0.19	1.09 (0.96-1.24)	0.0045	-0.06
rs11722786	55,169,182	C>T	0.366	0.357	0.22	1.08 (0.95-1.23)	0.0041	-0.10
rs11727002	55,169,183	A>G	0.366	0.357	0.22	1.08 (0.95-1.23)	0.0041	-0.10
rs55836714	55,170,352	T>C	0.384	0.374	0.19	1.09 (0.96-1.24)	0.0045	-0.07
rs77890222	55,170,462	C>A	0.366	0.357	0.22	1.08 (0.95-1.23)	0.0041	-0.10
rs62299407	55,171,129	A>G	0.366	0.357	0.22	1.08 (0.95-1.23)	0.0041	-0.10
rs4594778	55,172,289	C>T	0.366	0.357	0.22	1.08 (0.95-1.23)	0.0041	-0.10
rs4864877	55,172,357	A>G	0.366	0.357	0.22	1.08 (0.95-1.23)	0.0041	-0.10
rs13353653	55,172,713	G>A	0.366	0.357	0.22	1.08 (0.95-1.23)	0.0041	-0.10
rs4864878	55,173,715	C>T	0.366	0.357	0.22	1.08 (0.95-1.23)	0.0041	-0.10
rs6858442	55,173,933	G>A	0.366	0.357	0.22	1.08 (0.95-1.23)	0.0041	-0.10
rs6554168	55,174,827	G>A	0.366	0.357	0.22	1.08 (0.95-1.23)	0.0041	-0.10
rs6554170	55,174,885	C>T	0.366	0.357	0.22	1.08 (0.95-1.23)	0.0041	-0.10
rs1565668	55,175,601	T>C	0.366	0.357	0.22	1.08 (0.95-1.23)	0.0041	-0.10
rs1565665	55,175,768	C>G	0.366	0.357	0.22	1.08 (0.95-1.23)	0.0041	-0.10
rs28889275	55,176,073	G>A	0.366	0.357	0.22	1.08 (0.95-1.23)	0.0041	-0.10
rs28884174	55,176,383	T>C	0.366	0.357	0.22	1.08 (0.95-1.23)	0.0041	-0.10
rs28707743	55,178,018	C>T	0.364	0.356	0.23	1.08 (0.95-1.23)	0.0041	-0.11
rs28547147	55,179,059	C>A	0.365	0.357	0.22	1.08 (0.95-1.23)	0.0041	-0.10
rs10016845	55,183,077	T>C	0.364	0.356	0.23	1.08 (0.95-1.23)	0.0041	-0.11
rs11732117	55,184,119	A>G	0.364	0.356	0.23	1.08 (0.95-1.23)	0.0041	-0.11
rs6850695	55,185,035	C>T	0.364	0.356	0.23	1.08 (0.95-1.23)	0.0041	-0.11
rs10028483	55,186,168	T>C	0.364	0.356	0.23	1.08 (0.95-1.23)	0.0041	-0.11
rs9991681	55,186,390	G>A	0.364	0.356	0.23	1.08 (0.95-1.23)	0.0040	-0.12
rs28714526	55,186,508	A>G	0.364	0.356	0.23	1.08 (0.95-1.23)	0.0041	-0.11
rs2087806	55,186,956	C>T	0.364	0.356	0.23	1.08 (0.95-1.23)	0.0041	-0.11
rs10029431	55,187,406	A>T	0.364	0.356	0.23	1.08 (0.95-1.23)	0.0040	-0.12

SNP, single-nucleotide polymorphism; 1, major allele; 2, minor allele; OR, odds ratio; CI, confidence interval; PIP, posterior inclusion probability; $Log_{10}BF$, log_{10} Bayes factor.

Supplementary Table S4. Association and fine mapping results for 192 SNPs in the *PDGFRA* gene region in the cases with corneal astigmatism ≤ − 1.00 D

SNP	Position on Chr. 4 (GRCh37)	Alleles (1>2)	Minor Allele Freq. Cases (N=1134)	Controls (N=842)	*P*	*P*c	OR (95% CI)	Fine Mapping PIP	$Log_{10}BF$
rs17084040	55,074,368	G>A	0.125	0.127	0.90		1.01 (0.82-1.25)	0.00082	-0.81
rs17084042	55,074,542	G>T	0.125	0.127	0.90		1.01 (0.82-1.25)	0.00082	-0.81
rs57217483	55,074,922	G>T	0.125	0.127	0.90		1.01 (0.82-1.25)	0.00082	-0.81
rs28431840	55,075,792	T>G	0.125	0.127	0.90		1.01 (0.82-1.25)	0.00082	-0.81
rs6832597	55,076,237	C>A	0.209	0.178	0.021	0.19	1.23 (1.03-1.47)	0.0074	0.15
rs6831380	55,076,345	G>A	0.209	0.178	0.021	0.19	1.23 (1.03-1.47)	0.0074	0.15
rs7676972	55,077,078	C>T	0.209	0.178	0.021	0.19	1.23 (1.03-1.47)	0.0074	0.15
rs77086742	55,077,348	A>G	0.125	0.127	0.90		1.01 (0.82-1.25)	0.00082	-0.81
rs6554160	55,077,549	T>A	0.209	0.178	0.021	0.19	1.23 (1.03-1.47)	0.0074	0.15
rs111944671	55,078,108	G>A	0.219	0.200	0.12		1.14 (0.96-1.35)	0.0022	-0.38
rs28436914	55,078,194	A>T	0.219	0.200	0.12		1.14 (0.96-1.35)	0.0022	-0.38
rs28753397	55,078,496	C>T	0.219	0.200	0.12		1.14 (0.96-1.35)	0.0022	-0.38
rs139145626	55,078,614	C>T	0.209	0.178	0.021	0.19	1.23 (1.03-1.47)	0.0074	0.15
rs718454	55,079,544	G>C	0.219	0.200	0.12		1.14 (0.96-1.35)	0.0022	-0.38
rs718455	55,079,614	A>C	0.219	0.200	0.12		1.14 (0.96-1.35)	0.0022	-0.38
rs12648347	55,080,051	A>G	0.219	0.200	0.12		1.14 (0.96-1.35)	0.0022	-0.38
rs12505054	55,080,371	T>G	0.219	0.200	0.12		1.14 (0.96-1.35)	0.0022	-0.38
rs7673267	55,081,133	C>T	0.219	0.200	0.12		1.14 (0.96-1.35)	0.0022	-0.38
rs11133312	55,081,744	C>T	0.219	0.200	0.12		1.14 (0.96-1.35)	0.0022	-0.38
rs11133313	55,081,757	C>T	0.219	0.200	0.12		1.14 (0.96-1.35)	0.0022	-0.38
rs11133314	55,081,850	C>A	0.219	0.200	0.12		1.14 (0.96-1.35)	0.0022	-0.38
rs11133315	55,082,158	G>A	0.209	0.178	0.021	0.19	1.23 (1.03-1.47)	0.0074	0.15
rs4398585	55,082,353	C>T	0.219	0.200	0.12		1.14 (0.96-1.35)	0.0022	-0.38
rs28441665	55,083,056	T>C	0.125	0.127	0.90		1.01 (0.82-1.25)	0.00082	-0.81
rs4864855	55,083,451	G>T	0.219	0.200	0.12		1.14 (0.96-1.35)	0.0022	-0.38
rs4864856	55,084,720	G>A	0.219	0.200	0.12		1.14 (0.96-1.35)	0.0022	-0.38
rs7683707	55,086,036	T>C	0.219	0.200	0.12		1.14 (0.96-1.35)	0.0022	-0.38
rs60806423	55,086,816	C>T	0.188	0.156	0.012	0.11	1.26 (1.05-1.52)	0.011	0.32
rs192263827	55,087,113	T>C	0.103	0.111	0.091		0.83 (0.66-1.03)	0.0027	-0.29
rs113828936	55,087,144	G>A	0.188	0.156	0.012	0.11	1.26 (1.05-1.52)	0.011	0.32
rs17084051	55,087,581	C>A	0.188	0.156	0.012	0.11	1.26 (1.05-1.52)	0.011	0.32
rs28622224	55,088,093	C>T	0.313	0.283	0.031	0.28	1.18 (1.02-1.37)	0.0056	0.03
rs61320297	55,088,175	G>A	0.188	0.156	0.012	0.11	1.26 (1.05-1.52)	0.011	0.32
rs7673597	55,088,543	C>T	0.188	0.156	0.012	0.11	1.26 (1.05-1.52)	0.011	0.32
rs7673625	55,088,586	C>T	0.188	0.156	0.012	0.11	1.26 (1.05-1.52)	0.011	0.32
rs7673984	55,088,761	C>T	0.188	0.156	0.012	0.11	1.26 (1.05-1.52)	0.011	0.32
rs4394060	55,088,909	C>A	0.188	0.156	0.012	0.11	1.26 (1.05-1.52)	0.011	0.32
rs4864857	55,089,814	T>C	0.188	0.156	0.012	0.11	1.26 (1.05-1.52)	0.011	0.32
rs4864858	55,089,953	T>A	0.188	0.156	0.012	0.11	1.26 (1.05-1.52)	0.011	0.32
rs4864859	55,090,015	A>G	0.188	0.156	0.012	0.11	1.26 (1.05-1.52)	0.011	0.32
rs4864860	55,090,021	T>C	0.188	0.156	0.012	0.11	1.26 (1.05-1.52)	0.011	0.32
rs60863210	55,090,099	T>C	0.188	0.156	0.012	0.11	1.26 (1.05-1.52)	0.011	0.32
rs7698425	55,090,656	C>T	0.188	0.156	0.012	0.11	1.26 (1.05-1.52)	0.011	0.32
rs7681399	55,090,886	T>G	0.188	0.156	0.012	0.11	1.26 (1.05-1.52)	0.011	0.32
rs2114039	55,092,626	T>C	0.313	0.284	0.035	0.32	1.18 (1.01-1.37)	0.0051	-0.01
rs1800809	55,093,914	A>G	0.189	0.157	0.014	0.13	1.26 (1.05-1.51)	0.0095	0.26
rs6554162	55,093,955	G>A	0.313	0.284	0.035	0.32	1.18 (1.01-1.37)	0.0051	-0.01
rs1800810	55,094,031	C>G	0.189	0.157	0.014	0.13	1.26 (1.05-1.51)	0.0095	0.26
rs1800813	55,094,467	G>A	0.189	0.157	0.014	0.13	1.26 (1.05-1.51)	0.0095	0.26
rs1800812	55,094,629	G>T	0.189	0.157	0.014	0.13	1.26 (1.05-1.51)	0.0095	0.26
rs7690503	55,096,150	C>G	0.189	0.157	0.014	0.13	1.26 (1.05-1.51)	0.0095	0.26
rs7673027	55,096,180	T>A	0.189	0.157	0.014	0.13	1.26 (1.05-1.51)	0.0095	0.26
rs7668190	55,096,270	A>T	0.313	0.284	0.035	0.32	1.18 (1.01-1.37)	0.0051	-0.01
rs7689569	55,096,398	G>A	0.189	0.157	0.014	0.13	1.26 (1.05-1.51)	0.0095	0.26
rs7673853	55,096,606	T>C	0.189	0.157	0.014	0.13	1.26 (1.05-1.51)	0.0095	0.26
rs7679903	55,097,373	T>C	0.189	0.157	0.014	0.13	1.26 (1.05-1.51)	0.0095	0.26
rs890203	55,097,405	A>C	0.189	0.157	0.014	0.13	1.26 (1.05-1.51)	0.0095	0.26
rs4864861	55,097,685	C>T	0.189	0.157	0.014	0.13	1.26 (1.05-1.51)	0.0095	0.26
rs4864504	55,097,835	G>C	0.313	0.284	0.035	0.32	1.18 (1.01-1.37)	0.0051	-0.01
rs4608869	55,098,936	G>C	0.189	0.157	0.014	0.13	1.26 (1.05-1.51)	0.0095	0.26
rs4368668	55,098,954	A>G	0.189	0.157	0.014	0.13	1.26 (1.05-1.51)	0.0095	0.26
rs4635872	55,099,041	C>A	0.189	0.157	0.014	0.13	1.26 (1.05-1.51)	0.0095	0.26
rs6850748	55,099,164	T>G	0.189	0.157	0.014	0.13	1.26 (1.05-1.51)	0.0095	0.26
rs4864862	55,100,489	G>A	0.189	0.157	0.014	0.13	1.26 (1.05-1.51)	0.0095	0.26
rs4864863	55,100,831	A>G	0.189	0.157	0.014	0.13	1.26 (1.05-1.51)	0.0095	0.26
rs7678144	55,102,425	T>C	0.189	0.157	0.014	0.13	1.26 (1.05-1.51)	0.0095	0.26

Supplementary Table S4. (Cont.)

SNP	Position on Chr. 4 (GRCh37)	Alleles (1>2)	Minor Allele Freq.		*P*	*P* c	OR (95% CI)	Fine Mapping	
			Cases (N=1134)	Controls (N=842)				PIP	$Log_{10}BF$
rs6554163	55,102,559	T>A	0.189	0.157	0.014	0.13	1.26 (1.05-1.51)	0.0095	0.26
rs6836215	55,102,741	T>C	0.189	0.157	0.014	0.13	1.26 (1.05-1.51)	0.0095	0.26
rs67432867	55,104,454	A>T	0.189	0.157	0.014	0.13	1.26 (1.05-1.51)	0.0095	0.26
rs73252942	55,104,604	T>C	0.189	0.157	0.013	0.12	1.26 (1.05-1.51)	0.010	0.29
rs6554164	55,106,695	T>C	0.314	0.283	0.031	0.28	1.18 (1.01-1.37)	0.0055	0.03
rs35714192	55,107,750	G>C	0.459	0.437	0.29		1.08 (0.94-1.24)	0.0013	-0.61
rs147558377	55,113,623	C>G	0.187	0.156	0.013	0.12	1.26 (1.05-1.51)	0.010	0.29
rs13435164	55,115,828	C>G	0.313	0.283	0.032	0.29	1.18 (1.01-1.37)	0.0055	0.02
rs6554165	55,115,847	G>A	0.313	0.283	0.032	0.29	1.18 (1.01-1.37)	0.0055	0.02
rs79938737	55,116,048	G>C	0.124	0.126	0.87		1.02 (0.83-1.25)	0.00082	-0.80
rs60251664	55,117,805	T>C	0.124	0.126	0.87		1.02 (0.83-1.25)	0.00082	-0.80
rs76091088	55,119,631	T>C	0.103	0.111	0.091		0.83 (0.66-1.03)	0.0027	-0.29
rs4422471	55,121,921	C>T	0.187	0.154	0.012	0.11	1.27 (1.05-1.52)	0.011	0.32
rs10029499	55,122,494	A>G	0.312	0.281	0.028	0.25	1.18 (1.02-1.38)	0.0060	0.06
rs7677751	55,124,460	C>T	0.182	0.150	0.016	0.14	1.26 (1.04-1.51)	0.0088	0.23
rs9991165	55,124,591	A>G	0.178	0.146	0.013	0.11	1.27 (1.05-1.53)	0.010	0.30
rs2303429	55,124,870	C>T	0.103	0.111	0.091		0.83 (0.66-1.03)	0.0027	-0.29
rs2303430	55,125,058	A>T	0.462	0.471	0.80		1.02 (0.89-1.16)	0.00083	-0.80
rs73252946	55,125,992	G>A	0.176	0.146	0.018	0.16	1.25 (1.04-1.51)	0.0080	0.19
rs58435984	55,127,990	T>C	0.176	0.146	0.018	0.16	1.25 (1.04-1.51)	0.0080	0.19
rs2229307	55,130,078	T>C	0.176	0.146	0.018	0.16	1.25 (1.04-1.51)	0.0080	0.19
rs2307049	55,130,154	G>A	0.176	0.146	0.018	0.16	1.25 (1.04-1.51)	0.0080	0.19
rs67279506	55,130,682	A>G	0.176	0.146	0.018	0.16	1.25 (1.04-1.51)	0.0080	0.19
rs58727676	55,130,794	T>G	0.176	0.146	0.018	0.16	1.25 (1.04-1.51)	0.0080	0.19
rs7688997	55,131,450	C>A	0.176	0.146	0.018	0.16	1.25 (1.04-1.51)	0.0080	0.19
rs56145315	55,131,689	C>T	0.176	0.146	0.018	0.16	1.25 (1.04-1.51)	0.0080	0.19
rs28600756	55,131,732	C>A	0.176	0.146	0.018	0.16	1.25 (1.04-1.51)	0.0080	0.19
rs67600360	55,132,162	A>G	0.176	0.146	0.018	0.16	1.25 (1.04-1.51)	0.0080	0.19
rs4864864	55,132,325	C>T	0.176	0.146	0.018	0.16	1.25 (1.04-1.51)	0.0080	0.19
rs4864865	55,132,878	T>A	0.176	0.146	0.018	0.16	1.25 (1.04-1.51)	0.0080	0.19
rs73252948	55,133,252	T>C	0.176	0.146	0.018	0.16	1.25 (1.04-1.51)	0.0080	0.19
rs4358459	55,133,726	T>G	0.176	0.146	0.018	0.16	1.25 (1.04-1.51)	0.0080	0.19
rs28489067	55,133,936	C>T	0.176	0.146	0.018	0.16	1.25 (1.04-1.51)	0.0080	0.19
rs28650939	55,133,959	C>T	0.176	0.146	0.018	0.16	1.25 (1.04-1.51)	0.0080	0.19
rs28528897	55,134,339	C>G	0.176	0.146	0.018	0.16	1.25 (1.04-1.51)	0.0080	0.19
rs7660560	55,134,394	G>A	0.176	0.146	0.018	0.16	1.25 (1.04-1.51)	0.0080	0.19
rs7691129	55,134,466	T>C	0.176	0.146	0.018	0.16	1.25 (1.04-1.51)	0.0080	0.19
rs7686588	55,134,628	A>G	0.176	0.146	0.018	0.16	1.25 (1.04-1.51)	0.0080	0.19
rs12641563	55,135,447	C>G	0.176	0.146	0.018	0.16	1.25 (1.04-1.51)	0.0080	0.19
rs12644709	55,135,580	A>G	0.176	0.146	0.018	0.16	1.25 (1.04-1.51)	0.0080	0.19
rs12644749	55,135,771	A>G	0.176	0.146	0.018	0.16	1.25 (1.04-1.51)	0.0080	0.19
rs12508225	55,136,027	G>C	0.176	0.146	0.018	0.16	1.25 (1.04-1.51)	0.0080	0.19
rs12506290	55,136,132	T>A	0.176	0.146	0.018	0.16	1.25 (1.04-1.51)	0.0080	0.19
rs12505491	55,136,245	A>G	0.176	0.146	0.018	0.16	1.25 (1.04-1.51)	0.0080	0.19
rs1565669	55,137,183	T>C	0.176	0.146	0.018	0.16	1.25 (1.04-1.51)	0.0080	0.19
rs1565670	55,137,281	A>G	0.176	0.146	0.018	0.16	1.25 (1.04-1.51)	0.0080	0.19
rs35597368	55,139,771	T>C	0.163	0.139	0.047	0.42	1.21 (1.00-1.47)	0.0041	-0.10
rs869978	55,140,016	C>T	0.276	0.256	0.092		1.14 (0.98-1.33)	0.0026	-0.30
rs2307050	55,141,293	G>A	0.163	0.139	0.047	0.42	1.21 (1.00-1.47)	0.0041	-0.10
rs73252950	55,141,500	C>T	0.163	0.139	0.045	0.41	1.21 (1.00-1.47)	0.0042	-0.09
rs6855761	55,141,532	A>T	0.163	0.139	0.045	0.41	1.21 (1.00-1.47)	0.0042	-0.09
rs7656613	55,141,843	T>C	0.458	0.464	0.66		1.03 (0.90-1.18)	0.00088	-0.77
rs1105825	55,142,205	G>A	0.161	0.138	0.054		1.21 (1.00-1.46)	0.0037	-0.14
rs67388297	55,142,691	C>G	0.161	0.138	0.052		1.21 (1.00-1.46)	0.0038	-0.13
rs28698464	55,143,110	A>G	0.162	0.137	0.044	0.40	1.22 (1.00-1.47)	0.0043	-0.08
rs1316926	55,143,286	G>A	0.245	0.258	0.36		0.93 (0.80-1.09)	0.0011	-0.66
rs28374326	55,143,321	C>T	0.164	0.138	0.036	0.33	1.23 (1.01-1.49)	0.0049	-0.03
rs10028020	55,143,577	G>A	0.164	0.138	0.038	0.34	1.22 (1.01-1.48)	0.0048	-0.04
rs1907819	55,144,420	G>A	0.171	0.145	0.055		1.20 (1.00-1.45)	0.0037	-0.15
rs2412556	55,145,258	A>G	0.170	0.145	0.059		1.20 (0.99-1.44)	0.0035	-0.17
rs4289498	55,145,432	A>G	0.170	0.145	0.059		1.20 (0.99-1.44)	0.0035	-0.17
rs1547904	55,146,389	C>T	0.170	0.145	0.059		1.20 (0.99-1.44)	0.0035	-0.17
rs1547905	55,146,754	C>A	0.170	0.145	0.059		1.20 (0.99-1.44)	0.0035	-0.17
rs3816888	55,146,761	C>T	0.021	0.021	0.56		1.15 (0.72-1.85)	0.00094	-0.75
rs58025349	55,146,927	A>T	0.170	0.145	0.059		1.20 (0.99-1.44)	0.0035	-0.17
rs2291591	55,147,769	C>T	0.084	0.093	0.55		0.93 (0.73-1.18)	0.00094	-0.74
rs28811736	55,148,547	C>G	0.170	0.145	0.059		1.20 (0.99-1.44)	0.0035	-0.17

Supplementary Table S4. (Cont.)

SNP	Position on Chr. 4 (GRCh37)	Alleles (1>2)	Minor Allele Freq.		*P*	*P* c	OR (95% CI)	Fine Mapping	
			Cases (N=1134)	Controls (N=842)				PIP	$Log_{10}BF$
rs7677708	55,149,258	A>G	0.170	0.145	0.059		1.20 (0.99-1.44)	0.0035	-0.17
rs2412557	55,149,457	A>C	0.170	0.145	0.059		1.20 (0.99-1.44)	0.0035	-0.17
rs2412558	55,149,507	A>G	0.170	0.145	0.059		1.20 (0.99-1.44)	0.0035	-0.17
rs2228230	55,152,040	C>T	0.167	0.144	0.087		1.18 (0.98-1.43)	0.0027	-0.28
rs4864872	55,152,284	G>T	0.167	0.144	0.087		1.18 (0.98-1.43)	0.0027	-0.28
rs11940889	55,152,583	C>T	0.167	0.144	0.087		1.18 (0.98-1.43)	0.0027	-0.28
rs11931555	55,152,715	G>C	0.168	0.144	0.066		1.19 (0.99-1.44)	0.0033	-0.20
rs10020847	55,153,134	C>T	0.167	0.144	0.087		1.18 (0.98-1.43)	0.0027	-0.28
rs144143098	55,153,551	A>G	0.020	0.021	0.93		0.98 (0.61-1.58)	0.00082	-0.81
rs10021728	55,154,109	C>T	0.167	0.144	0.087		1.18 (0.98-1.43)	0.0027	-0.28
rs9993187	55,154,250	T>C	0.167	0.144	0.087		1.18 (0.98-1.43)	0.0027	-0.28
rs11733839	55,154,527	G>C	0.167	0.144	0.087		1.18 (0.98-1.43)	0.0027	-0.28
rs55732997	55,154,891	T>C	0.167	0.144	0.087		1.18 (0.98-1.43)	0.0027	-0.28
rs10004857	55,155,980	G>A	0.167	0.144	0.087		1.18 (0.98-1.43)	0.0027	-0.28
rs11133317	55,156,091	G>T	0.167	0.144	0.087		1.18 (0.98-1.43)	0.0027	-0.28
rs4864875	55,156,166	A>G	0.166	0.141	0.054		1.21 (1.00-1.46)	0.0037	-0.14
rs4864876	55,156,300	C>T	0.166	0.141	0.054		1.21 (1.00-1.46)	0.0037	-0.14
rs2276948	55,156,400	G>A	0.166	0.141	0.054		1.21 (1.00-1.46)	0.0037	-0.14
rs13147194	55,157,134	T>G	0.166	0.141	0.054		1.21 (1.00-1.46)	0.0037	-0.14
rs10010509	55,157,206	G>T	0.166	0.141	0.054		1.21 (1.00-1.46)	0.0037	-0.14
rs10003055	55,157,666	T>G	0.165	0.141	0.056		1.20 (0.99-1.46)	0.0036	-0.16
rs11935157	55,158,514	A>T	0.164	0.140	0.058		1.20 (0.99-1.46)	0.0036	-0.16
rs139471049	55,158,597	A>G	0.083	0.090	0.68		0.95 (0.74-1.21)	0.00087	-0.78
rs60218083	55,158,938	A>G	0.163	0.139	0.059		1.20 (0.99-1.46)	0.0035	-0.17
rs55784333	55,159,391	C>G	0.163	0.139	0.059		1.20 (0.99-1.46)	0.0035	-0.17
rs10032688	55,160,658	G>A	0.163	0.139	0.059		1.20 (0.99-1.46)	0.0035	-0.17
rs3733540	55,161,254	T>C	0.163	0.139	0.059		1.20 (0.99-1.46)	0.0035	-0.17
rs3690	55,161,813	A>C	0.163	0.139	0.059		1.20 (0.99-1.46)	0.0035	-0.17
rs17739921	55,164,866	A>C	0.275	0.285	0.32		0.93 (0.80-1.08)	0.0012	-0.63
rs34491905	55,165,075	C>T	0.274	0.283	0.37		0.93 (0.80-1.08)	0.0011	-0.66
rs1961871	55,167,266	G>A	0.368	0.357	0.13		1.11 (0.97-1.28)	0.0021	-0.40
rs1826426	55,167,287	A>G	0.416	0.403	0.061		1.14 (0.99-1.31)	0.0035	-0.18
rs10000267	55,167,931	A>G	0.387	0.374	0.086		1.13 (0.98-1.30)	0.0028	-0.28
rs11722786	55,169,182	C>T	0.368	0.357	0.13		1.11 (0.97-1.28)	0.0021	-0.40
rs11727002	55,169,183	A>G	0.368	0.357	0.13		1.11 (0.97-1.28)	0.0021	-0.40
rs55836714	55,170,352	T>C	0.387	0.374	0.088		1.13 (0.98-1.30)	0.0027	-0.28
rs77890222	55,170,462	C>A	0.368	0.357	0.13		1.11 (0.97-1.28)	0.0021	-0.40
rs62299407	55,171,129	A>G	0.368	0.357	0.13		1.11 (0.97-1.28)	0.0021	-0.40
rs4594778	55,172,289	C>T	0.368	0.357	0.13		1.11 (0.97-1.28)	0.0021	-0.40
rs4864877	55,172,357	A>G	0.368	0.357	0.13		1.11 (0.97-1.28)	0.0021	-0.40
rs13353653	55,172,713	G>A	0.368	0.357	0.13		1.11 (0.97-1.28)	0.0021	-0.40
rs4864878	55,173,715	C>T	0.368	0.357	0.13		1.11 (0.97-1.28)	0.0021	-0.40
rs6858442	55,173,933	G>A	0.368	0.357	0.13		1.11 (0.97-1.28)	0.0021	-0.40
rs6554168	55,174,827	G>A	0.368	0.357	0.13		1.11 (0.97-1.28)	0.0021	-0.40
rs6554170	55,174,885	C>T	0.368	0.357	0.13		1.11 (0.97-1.28)	0.0021	-0.40
rs1565668	55,175,601	T>C	0.368	0.357	0.13		1.11 (0.97-1.28)	0.0021	-0.40
rs1565665	55,175,768	C>G	0.368	0.357	0.13		1.11 (0.97-1.28)	0.0021	-0.40
rs28889275	55,176,073	G>A	0.368	0.357	0.13		1.11 (0.97-1.28)	0.0021	-0.40
rs28884174	55,176,383	T>C	0.368	0.357	0.13		1.11 (0.97-1.28)	0.0021	-0.40
rs28707743	55,178,018	C>T	0.366	0.356	0.14		1.11 (0.97-1.28)	0.0020	-0.42
rs28547147	55,179,059	C>A	0.367	0.357	0.13		1.11 (0.97-1.28)	0.0021	-0.40
rs10016845	55,183,077	T>C	0.366	0.356	0.14		1.11 (0.97-1.28)	0.0020	-0.42
rs11732117	55,184,119	A>G	0.366	0.356	0.14		1.11 (0.97-1.28)	0.0020	-0.42
rs6850695	55,185,035	C>T	0.366	0.356	0.14		1.11 (0.97-1.28)	0.0020	-0.42
rs10028483	55,186,168	T>C	0.366	0.356	0.14		1.11 (0.97-1.28)	0.0020	-0.42
rs9991681	55,186,390	G>A	0.366	0.356	0.15		1.11 (0.96-1.28)	0.0019	-0.43
rs28714526	55,186,508	A>G	0.366	0.356	0.14		1.11 (0.97-1.28)	0.0020	-0.42
rs2087806	55,186,956	C>T	0.366	0.356	0.14		1.11 (0.97-1.28)	0.0020	-0.42
rs10029431	55,187,406	A>T	0.366	0.356	0.15		1.11 (0.96-1.28)	0.0019	-0.43

SNP, single-nucleotide polymorphism; 1, major allele; 2, minor allele; *P* c, corrected *P* -value; OR, odds ratio; CI, confidence interval; PIP, posterior inclusion probability; $Log_{10}BF$, log_{10} Bayes factor.

Supplementary Table S5. Functional annotation of the lead SNPs in the *PDGFRA* gene region identified in this study.

SNP	Position on Chr. 4 (GRCh37)	HaploReg: Promoter histone marks	HaploReg: Enhancer histone marks	HaploReg: DNAse	HaploReg: Proteins bound	HaploReg: Transcription factor motifs altered	RegulomeDB: Score[a]
Lead SNPs in the cases with corneal astigmatism ≤ − 1.50 D							
rs6832597	55,076,237	-	-	-	-	GR, HDAC2, p300	5
rs6831380	55,076,345	-	-	-	-	-	7
rs7676972	55,077,078	-	-	-	-	Myc, Myf	5
rs6554160	55,077,549	-	-	-	-	Cart1, Foxa, Foxp1, HDAC2, HNF1, Sox, Zfp105, p300	6
rs139145626	55,078,614	-	-	-	-	ERalpha-a, Nrf-2, TCF11::MafG	7
rs11133315	55,082,158	-	5 tissues[b]	SKIN	-	Foxp1	4
Lead SNPs in the cases with corneal astigmatism ≤ − 1.25 D							
rs60806423	55,086,816	-	-	-	-	Brachyury, E2A, LBP-1	5
rs113828936	55,087,144	-	-	-	-	BDP1, Maf, NF-E2	5
rs17084051	55,087,581	-	-	-	-	-	5
rs61320297	55,088,175	-	-	-	-	STAT	7
rs7673597	55,088,543	-	-	-	-	CACD, Klf4, Klf7, NRSF, SP1, Znf143	5
rs7673625	55,088,586	-	-	-	-	Irf	4
rs7673984	55,088,761	-	-	-	-	PLZF	5
rs4394060	55,088,909	-	-	-	-	EWSR1-FLI1, GATA, HDAC2, TATA	5
rs4864857	55,089,814	-	-	-	-	CTCF, Rad21, ZBTB33	7
rs4864858	55,089,953	-	-	-	-	42 altered motifs	6
rs4864859	55,090,015	-	-	-	-	AhR, CHOP::CEBPalpha, Maf, SREBP, Zbtb3	5
rs4864860	55,090,021	-	-	-	-	E2F, SREBP, YY1	5
rs60863210	55,090,099	-	-	-	-	-	5
rs7698425	55,090,656	-	4 tissues[c]	-	-	AP-1, BDP1, CHD2, Irf, Klf4, NRSF, Pax-5, SP1, STAT, TATA, ZEB1	5
rs7681399	55,090,886	-	5 tissues[d]	-	-	HNF4, LF-A1, Nr2f2	5

[a]The RegulomeDB score ranges from 1a to 7, with lower scores indicating an increased likelihood of having a regulatory function. Scores 4, 5, and 6: minimal evidence for regulatory function; score 7: no annotation data available.

[b]ESC (embryonic stem cells), ESDR (embroyonic stem cell-derived), FAT (adipose-derived mesenchymal stem cells), SKIN (skin), and LNG (lung).

[c]FAT, SKIN, MUS (Muscle), and GI (gastrointestinal tract).

[d]ESC, FAT, SKIN, MUS, and GI.
